# NetSeekR: a network analysis pipeline for RNA-Seq time series data

**DOI:** 10.1186/s12859-021-04554-1

**Published:** 2022-01-28

**Authors:** Himangi Srivastava, Drew Ferrell, George V. Popescu

**Affiliations:** 1 Department of Electrical and Computer Engineering, Mississippi State University, Mississippi State, MS 39762 USA; 2 Department of Biochemistry, Molecular Biology, Plant Pathology and Entomology, Mississippi State University, Mississippi State, MS 39762 USA; 3 Institute for Genomics, Biocomputing and Bioengineering, Mississippi State University, Mississippi State, MS 39762 USA

**Keywords:** RNA-Seq data, Differential gene expression analysis, Correlation gene expression analysis, Regulatory network analysis, Complex network analysis, Bioinformatics pipeline

## Abstract

**Background:**

Recent development of bioinformatics tools for Next Generation Sequencing data has facilitated complex analyses and prompted large scale experimental designs for comparative genomics. When combined with the advances in network inference tools, this can lead to powerful methodologies for mining genomics data, allowing development of pipelines that stretch from sequence reads mapping to network inference. However, integrating various methods and tools available over different platforms requires a programmatic framework to fully exploit their analytic capabilities. Integrating multiple genomic analysis tools faces challenges from standardization of input and output formats, normalization of results for performing comparative analyses, to developing intuitive and easy to control scripts and interfaces for the genomic analysis pipeline.

**Results:**

We describe here NetSeekR, a network analysis R package that includes the capacity to analyze time series of RNA-Seq data, to perform correlation and regulatory network inferences and to use network analysis methods to summarize the results of a comparative genomics study. The software pipeline includes alignment of reads, differential gene expression analysis, correlation network analysis, regulatory network analysis, gene ontology enrichment analysis and network visualization of differentially expressed genes. The implementation provides support for multiple RNA-Seq read mapping methods and allows comparative analysis of the results obtained by different bioinformatics methods.

**Conclusion:**

Our methodology increases the level of integration of genomics data analysis tools to network inference, facilitating hypothesis building, functional analysis and genomics discovery from large scale NGS data. When combined with network analysis and simulation tools, the pipeline allows for developing systems biology methods using large scale genomics data.

**Supplementary Information:**

The online version contains supplementary material available at 10.1186/s12859-021-04554-1.

## Background

Multiple RNA-Seq data analysis pipelines of increased analytic performance have been developed over the past decade, prompted by the availability of NGS data (i.e. STAR [1], HISAT [2]). The pipelines, typically running on large scale computing platforms available in research centers or on cloud computing servers, provide data summarization from millions of reads to thousands of gene expression estimates as well as statistical analysis of gene expression changes associated with experimental treatments (edgeR [3], DESeq2 [4]). Alternative faster pipelines have been developed recently, consisting of pseudo-aligners or alignment-free tools (Kallisto [5], Salmon [6]) and associated statistical data analysis platforms (i.e. Sleuth [7]). With the increasing performance of the RNA-Seq data analysis tools comes the opportunity to create larger scale designs that include temporal dynamics of observed biological processes, the possibility to evaluate multiple pipelines for optimal decisions on gene expression analyses and the capacity to integrate network biology tools to analyze gene expression dynamics. Given the large variation in performance between aligners as well as their dependencies on data [8], there is a need to compare the results from multiple software tools in order to optimize the processing of RNA-Seq data. Here we provide an integration of one of the best performing spliced aligners—STAR—with a pseudo-aligner—Kallisto—as well as two differential gene expression analysis tools (edgeR and Sleuth) using different statistical models and data analysis and visualization methods.

A typical RNA-Seq data analysis pipeline consists of data preprocessing (quality control of sequencing data, reads trimming), reads mapping and gene expression quantification. This is typically followed by an assessment of differentially expressed genes with the objective of evaluating factors that control gene transcription. The statistical analysis is derived from the experimental design that describes treatment and control samples. In addition to identifying sets of differentially expressed genes, subsequent analyses aim at using functional and cell system information in order to identify networks and pathways associated with treatments. In this paper we present NetSeekR, an RNA-Seq data analysis R package aimed at analyzing the transcriptome dynamics for inferring networks of differentially expressed genes associated with experimental treatments measured at multiple time points.

## Implementation

The core software pipeline we present here performs alignment of reads, differential gene expression analysis, gene ontology enrichment analysis and network analysis of differentially expressed genes. Along with the pipeline, we include various parameter setups and exemplify two different methodologies for differential gene expression analysis. The input of the pipeline are sets of files containing raw reads from the high throughput sequencing step. These reads were previously processed with a quality control check using FastQC [9] and trimmed using Trimmomatic [10] software. The first step of the pipeline is the alignment of processed reads to genome positions using the gene annotation file. NetSeekR currently implements two read mapping tools—STAR and Kallisto—in order to allow comparative evaluation of transcript quantification. The next step in the pipeline is the identification of differentially expressed genes. The data obtained in the previous step is subset for mapping counts data to conditions, loaded into an R data frame and converted into matrix format. Two options are available at this stage of the pipeline—edgeR [3] and Sleuth [7]—offering two options for gene expression modeling, complementary methods for identification of differentially expressed genes and multiple methods for data normalization and visualization. Both software tools implement statistical methodologies for carrying out various operations on gene counts data such as filtering, normalization, multidimensional plotting, and clustering.

The next stage of the pipeline aims to use statistical tools to predict gene networks and to compute functional overrepresentation of differentially expressed genes obtained in the previous step. We have currently included two methods to create and analyze correlated gene expression networks and to infer regulatory networks. The aim of gene network analysis is to identify pathways associated with the experimental treatment by mining differential gene expression patterns. To accomplish this task, we use the Weighted Correlation Network Analysis (WGCNA) [11] to identify patterns or clusters in gene expression data and the Dynamic Regulatory Events Miner (DREM) [12] to identify regulatory patterns that drive the observed gene expression. The NetSeekR pipeline uses the functionality provided by WGCNA package to perform network construction, module detection and topological overlap matrix construction of gene expression. The output of the pipeline can be used to find out biologically interesting modules by correlating gene expression changes with phenotype changes when provided by the experimental design. The pipeline also uses GO enrichment analysis to mine the functionality of gene expression data on selected sets of genes identified in our analysis. The pipeline implements DREM to infer regulatory networks from time series of gene expression or from series of treatments and/or genotype variation data. Finally, the *igraph* [13] and *tidygraph* [14] R packages are used to conduct network analysis on the differentially expressed gene (DEG) networks using custom scripts, for mapping overlapping nodes between DEG sets and gene networks from public data sources and for visualization of network analyses results.

The pipeline processing begins by reading in several arguments from a configuration file and making a directory tree to store data. Arguments to the pipeline include: a string specifying the analysis type, covariates to account for differential testing, a path to a differential gene expression sample comparison matrix (DGECM), a path to an experimental design matrix on which DGECM is based, parameters for differential gene expression analysis (a significance level for statistical testing of differential gene expression), a path to a directory containing raw read sequences, a path to a selected output directory, a path to a reference genome, and Boolean flags for specifying whether to implement Kallisto or STAR pipelines, or both. The directory tree is structured such that the current working directory is the top-most node, with subdirectories within the tree for DREM, edgeR, Kallisto, network analysis, Sleuth, and WGCNA data.

The first two steps of the pipeline consist of building a transcriptome index and quantifying reads. Both STAR and Kallisto operations are executed from the R environment through the Linux terminal by passing a bash script, assembled from arguments. Arguments are passed to NetSeekR from the configuration file to match the type of pipeline (STAR or Kallisto) being executed. The R-implemented bash scripts for index-building operations use a genome annotation file reference in the configuration file (using the path as an input argument) to generate the transcriptome index for Kallisto/STAR in the specified data storage directory created in the directory tree.

The read quantification step is also directed to the Linux terminal from within the R code. The quantification commands sent to the terminal are concatenated together in a bash script using variables given to the function from the configuration file. The bash script for running the STAR/Kallisto quantification method is called from within the R environment. The STAR/Kallisto quantification method creates a directory for each sample quantified with directory names derived from sample identifiers. Arguments to the command include: the shell script name, the read data directory path, the path to the Kallisto or STAR index file, a directory path for storing quantification results, and the log file directory path. Running a bash script from within the R environment has the advantage that large datasets (mRNA sequencing read data files) do not need to be loaded into the memory of R, saving time and avoiding memory size issues.

Quantification data and the design matrix are both accessed in the next step for differential gene expression (DGE) computation. The experimental design matrix (DM) is a file which consists of paths to samples, and respective characteristics defined in the experimental design prior to sequencing. The experimental design file is string-processed to provide a dataset with references to variables for DGE software to use. The design matrix used as input has to conform to a specified format and can be edited with a file editing program. The DM is used in tandem with the DGECM supplied to the pipeline; the DGECM groups together the samples from the DM to be compared when testing for differential gene expression. The DGECM columns are combined together in the R code row-wise with a ‘logical or’ string between each sample identifier to select test samples. The number of rows in the DGECM corresponds to the total number of sample comparisons in the analysis, each row corresponding to one comparison instance. The cells contain sample identifiers matching the samples described in the experimental design file.

**Fig. 1:**
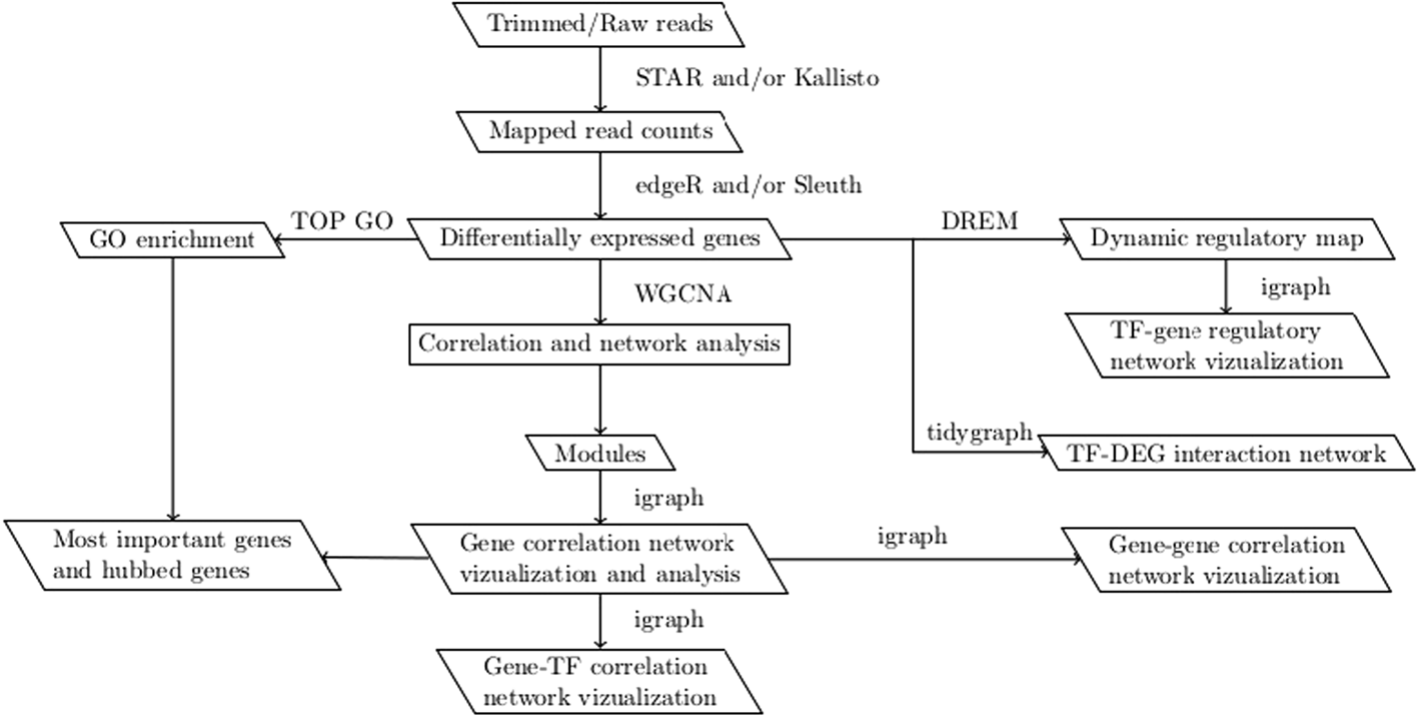
Workflow of the NetSeekR pipeline

The last component of the pipeline is network analysis of the data. This involves processing of WGCNA input (differentially expressed genes obtained from edgeR/Sleuth and their estimated expression values) to generate correlation networks. Next, we carry out GO enrichment analysis on same set of rearranged data using *topGo* software [15]. The last step is network construction and visualization using *igraph*. The pipeline’s workflow is shown in Fig. 1.

Framework extension may be conducted with NetSeekR such that other packages can be added to those pre-existing in the framework. Several data structures used to analyze networks are available to the user for accessing. These data structures can be extracted from within the network analysis functions we have implemented. The way NetSeekR was developed makes it easy for the user to access calculations made when analyzing networks through tibble data structures [16]. Framework extension therefore should align with these structures since mapping functions over columns/variables is made simple. The additional functions could be made available through other installed packages. For example, clustering variables such as distance and adjacency matrices, can be accessed from the tibble structure returned from the expr2 variable in the *implement_network_analysis* function. The Gene-TF network analysis we conduct is controlled in the same fashion, i.e., the procedure is written and then used on batched datasets. Code can be written to implement a new procedure, or installed function, and then plugged into NetSeekR by making use of the existing columns (including separate differential expression datasets) in the expr2 variable. The details of pipeline implementation are explained in the next sections.

### Reads mapping and differentially gene expression analysis

#### STAR and edgeR

Our pipeline implementation uses STAR (Spliced Transcripts Alignment to a Reference) aligner software to carry out the read alignment of RNA-Seq time series data. The input to the STAR aligner consists of reference genome and the annotation file which need to be downloaded or accessed from public databases using tools such as biomaRt [17]. STAR alignment consists of two steps: (1) generating genome indexing from target genome annotation files and (2) alignment of reads contained in FASTAQ files using genome indexing information. After mapping the FASTAQ file to the reference genome, STAR stores the result of the alignment file in a SAM (Sequencing Alignment mapping read) or BAM (Binary alignment mapping reads) format [18] in the pipeline-specified directories. FeatureCounts software [19] summarizes then the reads counts mapped from the RNA sequencing files in the previous task. Next the workflow uses edgeR pipeline for differential gene expression analysis. The RNA -seq pipeline uses various statistical methodologies available in this package for carrying out filtering, normalization, multidimensional plotting, identification of genes with significant expression changes, clustering etc. A typical processing in edgeR includes the following steps: (1) loading the data as an R data frame and then combine into a matrix where each cell in a matrix represents the count of the number of the reads for a particular gene; (2) scaling and normalization of counts per million (CPM) and Log counts per million (Log-CPM) data; (3) filtering out the genes that are not expressed; (4) removing any data artifacts or batch effect in the using robust normalization of the data (we use edgeR trimmed mean of M-value (TMM) method normalization); (5) creating a DGEList object to hold the read counts that remains after the filtering; (6) plotting multidimensional scaling plots to perform the analysis the inter-sample relationships; (7) setting up the model by taking the DGEList object and the groups and formed the design matrix; (8) using *voom* to perform additional data normalization to remove batch effect and other processing biases; (9) forming the contrast matrix for the analysis of the comparison of interest; (10) model data fitting—this step is carried out using *Limma* function *lmfit* and contrast.fit for contrast matrix; empirical Bayes moderation can be applied to contrast matrix; (11) using *treat* method of *Treat* function provided by *Limma* package [20] to set log-FC value requirements. The *treat* method allows to set decisions based on *p value* results from the empirical Bayes moderator t-statistics and the target log-FC threshold; (12) examining differentially expressed data partitioned into up regulated and downregulated genes and storing them in “.csv” files for each of comparison; generating the mean difference plot to study the upregulated and downregulated genes.

#### Kallisto and Sleuth

Kallisto is a command-line tool used for quantifying transcript abundances by pseudo-alignment. An R script was designed to pseudo-align, using Kallisto, batches of trimmed mRNA reads (according to the sample processing configuration file), and to test, using Sleuth, for differentially expressed genes. The pipeline script uses as input a reference genome annotation file and the mRNA read datasets. Three other input files are also necessary: one file containing an edited design matrix, another with arguments to the pipeline and a third indicating sample comparisons to make when testing for potential differentially expressed genes. Genome annotations files can be imputed either through the biomaRt R package or provided as input after download from a public database. One downside to the biomaRt gene extraction method is that some annotations might not be up to date (i.e. for A. thaliana currently only the TAIR10 annotation is available, and not Araport11 [21]). One advantage to using biomaRt is that annotations are available for each gene so descriptions can be returned in the differential expression analysis alongside statistics for each gene.

To perform differential gene expression analysis, Sleuth is programed to iterate over the comparison sets specified in the DGECM file. Sleuth provides both transcript- and gene-level differential gene expression analysis functionality. A Boolean flag is written in the arguments to specify the analysis level. The code captures each of three cases: transcript-level, gene-level, or both. Gene-level analysis requires an additional dataset with transcript identifiers mapped to corresponding gene names. Alternatively, Kallisto count data can be analyzed in edgeR after a data transformation involving gene isoform removal.

### Network inference

#### Gene correlation networks analysis

NetSeekR implements statistical network analysis using WGCNA which includes network construction, module detection, and topological overlap matrix construction of the gene expression data. WGCNA (Weighted Gene Correlation Network Analysis) is an R software package used to infer gene networks from transcriptomics data by applying topological constraints derived from statistical analysis of complex networks. It includes additional methods for integration of phenotype or traits data with the gene expression data. In WGCNA, highly correlated genes clusters called modules are identified using a hierarchical clustering technique. We also compute module eigengenes, the first principle component representing a gene expression profile of a given module, and assess the proportion of variance among the genes in a particular module.

In the NetSeekR pipeline, we first perform a data preprocessing step consisting of filtering of lowly expressed genes from the output of Sleuth/edgeR. To carry out the further analysis we also filter out the genes with an excessive number of missing samples. We then visualize the clustering of the samples using an Euclidean distance measure. The gene correlation network is specified by the adjacency matrix, obtained from a similarity matrix which measure the level of similarity between the gene expression profiles across all the samples. The similarity matrix contains the absolute value of correlation between series of expression data for each pair of genes in the dataset. For large-scale experimental designs, we construct these series to span time points, treatments and conditions, genotypes etc. We use Pearson correlation to calculate the similarity of pairs of genes in our dataset. An adjacency function transforms the similarity matrix containing co-expression similarities into the adjacency matrix containing connection strengths. A typical choice is biweight midcorrelation (bicor) as it is more robust and less susceptible to outliers while capturing monotonic relations between genes. We use the soft thresholding measure to set the adjacency matrix power. Our implementation uses the *pickSoftthreshold* function that performs the analysis of network topology in order to select the appropriate soft threshold. Next, the Topological Overlap Matrix (TOM), a measure of connectivity strength between the two nodes based on the overlap of common neighbors, is computed. After calculating the overlap matrix, the next step is module detection. The *Modules* are the clusters of closely interconnected nodes which are genes with high topological overlaps obtained using the unsupervised clustering technique provided by the WGCNA package. Our pipeline performs average linkage hierarchical clustering using the dissimilarity matrix obtained from Topological Overlap Matrix. We obtain a cluster dendrogram of all the genes using the R function *hclust*: in the cluster dendrogram the modules are detected as branches and dynamic tree cut is used to perform module identification. A further step is the reduction of the number of modules resulted after clustering. We first quantify the co-expression similarity of modules by calculating the eigengenes and clustering them based on their correlation. We then merge the modules with the similar expression profile. For module display we use the TOM plot, which is a summary of the co-expression network, showing the values of the dissimilarity matrix.

#### Gene regulatory networks (GRN) analysis

To infer GRNs, *w*e use *c*ount data from the read mapping pipelines as input for DREM. First, we perform a dataset rearrangement that ensures the data series structure associates with the GRN analysis design (typically series span time points and/or treatments). Networks are graphed for differential expression gene sets to show overlap between an input network (default is the DREM input network) and the differentially expressed genes; mean read counts per gene are also represented. Count data from the pseudo-alignment performed by STAR/Kallisto is used to produce DREM input data. If needed, reads for multiple gene models are aggregated together by summing count data for each individual gene. Aggregated gene quantification sets are saved to unique files based on sample identifiers in a data directory (from the directory tree). A data transformation is implemented on the edited quantification data (aggregate read counts) such that read counts are arranged in a time series format for each condition and each replicate. The time series rearrangement results are stored in uniquely named files in a directory to be accessed by DREM. DREM is executed for each dataset corresponding to a time series for a genotype, condition and technical replicate. Time series datasets discussed prior is accessed iteratively for analysis; the argument defaults file provided by DREM2.0 (defaults.txt) is edited in the R code by inserting paths to the time series datasets into the proper cells, accounting for technical replicates. All arguments to DREM other than those used for iterating over time series datasets need to be specified in the defaults template file prior to running the pipeline. NetSeekR calls an instance of the DREM GUI for each genotype, condition and technical replicate dataset. Files generated for DREM input should be used in the DREM GUI. Gene tables for each path should be saved, once DREM finishes executing, to the DREM analysis-specific directory with a literal ‘path’ string to be extracted in network analysis.

### Gene ontology analysis

After identifying the WGCNA modules, we perform the GO enrichment analysis for each of the modules obtained in our analysis from DEG sets. The aim is to identify significantly overrepresented gene categories and biological processes associated with the treatment performed in experimental design. Since the GO package included with WGCNA (*anRichment* [22]) does not provide support for many genomes (including plant genomes), we have added the package *topGO* [15] to perform this analysis. Using *topGO*, we tested overrepresentation of GO terms using a Kolmogorov–Smirnov test for GO enrichment of DEG genes.

### Network analysis

Further network analysis is provided using the *igraph* R package. One network analysis method consists in searching for overlapping nodes between differential gene expression sets and an existing gene network from public data sources. For example, such a network file is provided by DREM for six organisms. Other networks can be obtained from public databases such as: GeneMania [23], IntAct [24], STRING [25] and KEGG [26]. Further, count data derived from Kallisto or STAR results can be used to visualize average counts per gene with regard to the replicates belonging to each differential gene expression comparison set. Beta coefficients from Sleuth, or log fold changes from edgeR can also be visualized per gene in each extracted network. The processing of input files proceeds with reducing multiple sources and multiple targets (gene identifiers) to generate unique edges. Initially, edges in the network file may contain multiple unique and non-unique gene nodes. Reduction ensures edges are unique, leaving one edge per row in the dataset. Differentially expressed gene (DEG) sets are then overlapped with nodes in the provided network, generating sets of nodes which are differentially expressed in each comparison set. Read counts of DEG genes are represented over each DEG network and per gene statistics are computed. Overlapping nodes are visualized in graphs where positive or negative beta coefficients are represented as distinct shapes, estimated counts are represented as size, and beta coefficients (scaled to a range from 0 to 1) are represented by shape opaqueness. When using input from Sleuth, beta coefficients for multiple gene isoforms are averaged; alternatively, Sleuth analysis can be performed for genes instead of gene models. EdgeR analysis produces log-fold-change information that can be visualized of the target networks using this package.

Metrics can be collected to characterize network structures and to provide insight into key pathways associated with experimental treatment. Node degree, centrality, shortest pathways, diameter, clustering coefficient etc. can be calculated on differential gene expression graphs. To illustrate these capabilities, we have implemented a method to generate networks of differentially expressed genes and to identify the hub genes of each comparison set. We calculated the node centrality score for all the clusters and identified the hub genes. We merged the hub genes obtained from each cluster to find top genes from the network. The list of top genes was then intersected with the most significant GO enrichment genes calculated using Kolmogorov–Smirnov testing in topGO package. To visualize network information, we incorporated the modules information obtained from WGCNA (cluster membership information of each gene) and combined it with the nodes(genes) data of the network data frame, assigning each nodes the color of the module to which that particular gene belonged to. The functions implemented by NetSeekR are briefly described in Additional file 1: Table S1. The NetSeekR code and tutorial are included in the Additional file 2.

## Results and discussion

**Fig. 2:**
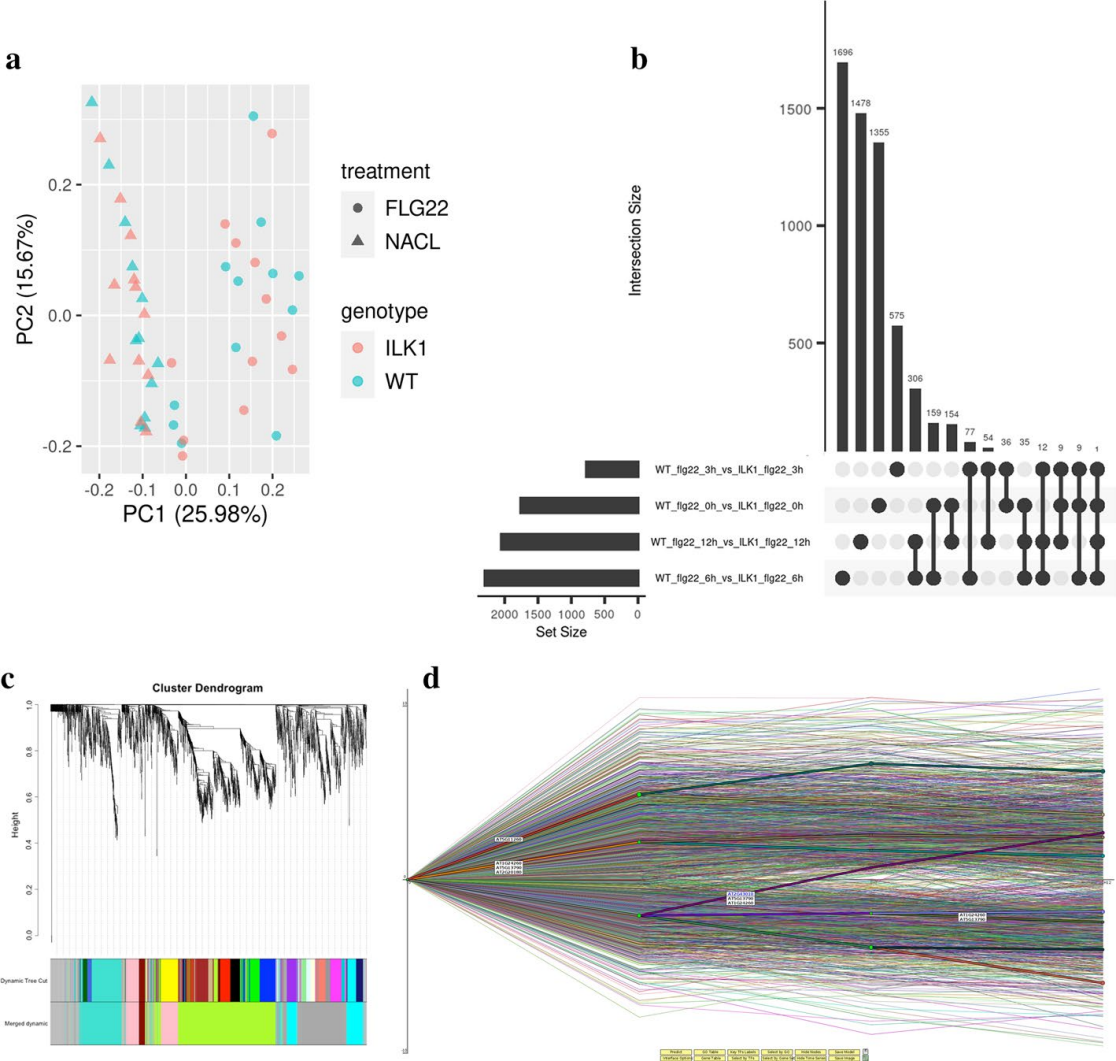
**a** PCA plot from Sleuth analysis of time-series of RNA-Seq data; **b** UpSetR comparison of time series of RNA-Seq data; **c** dendrogram from WGCNA analysis of time series of RNA-Seq data; **d** DREM analysis of time series of RNA-Seq data

NetSeekR allows integrated management of the RNA-Seq tools, comparative analysis of gene expression implemented using multiple pipelines and network analysis. It can provide support for sample comparison using PCA and MDS analyses, comparative analysis of DEG gene sets using UpSetR [27], visualization and analysis of gene correlation networks and visualization and analysis of gene regulatory networks. In Fig. 2 we enumerate visualization capabilities related to network analysis for an experimental design that includes timeseries data of four selected timepoints, two stress factors and two genotypes. Comparisons on statistics of gene expression estimates between a spliced read aligner and a pseudo-aligner can be used to better understand the dynamics of gene expression. The PCA and MDS plots show the sample correlation and its dynamics as a function of treatment and genotype as calculated with STAR-edgeR (SE) and Kallisto-Sleuth (KS) pipelines respectively (a sample PCA plot is shown in Fig. 2A). The UpSetR analysis allows DEG comparison between the SE and KS pipelines (Fig. 2B).

Next, we perform construction of correlation networks in order to analyze the dynamics of differentially expressed genes and to correlate this dynamic with observed phenotypes. Parametrization of WGCNA controls the scale free topology constraints that shape the structure of the correlation network. In addition, network constraints derived from publicly available interactome data can be superimposed to the resulting topology using network analysis software.

Figure 2C shows an example of a cluster dendrogram created by the average linkage hierarchical clustering obtained after merging the modules with similar co-expression profiles. Further development of the network analysis package includes graph motif frequency calculation across various sequences of data with regard to genotypes, time points or conditions. Methods to analyze sub-networks formed across gene-sets in series of time points, or across networks of genes that are differentially expressed can also be designed using this R package. Another dimension of analysis provided by our pipeline is represented by the regulatory network analysis. Figure 2D illustrates the dynamics of driver TF genes in the time series design with two treatments and two genotypes obtained with DREM 2.0.

Further network analysis performed with the *igraph* and *tidygraph* packages allows the creation of network objects, computation of statistics describing network structure (node degree distribution, network clustering, betweenness, closeness, and eigenvector centrality and identification of network hubs) as well as network visualization.

**Fig. 3:**
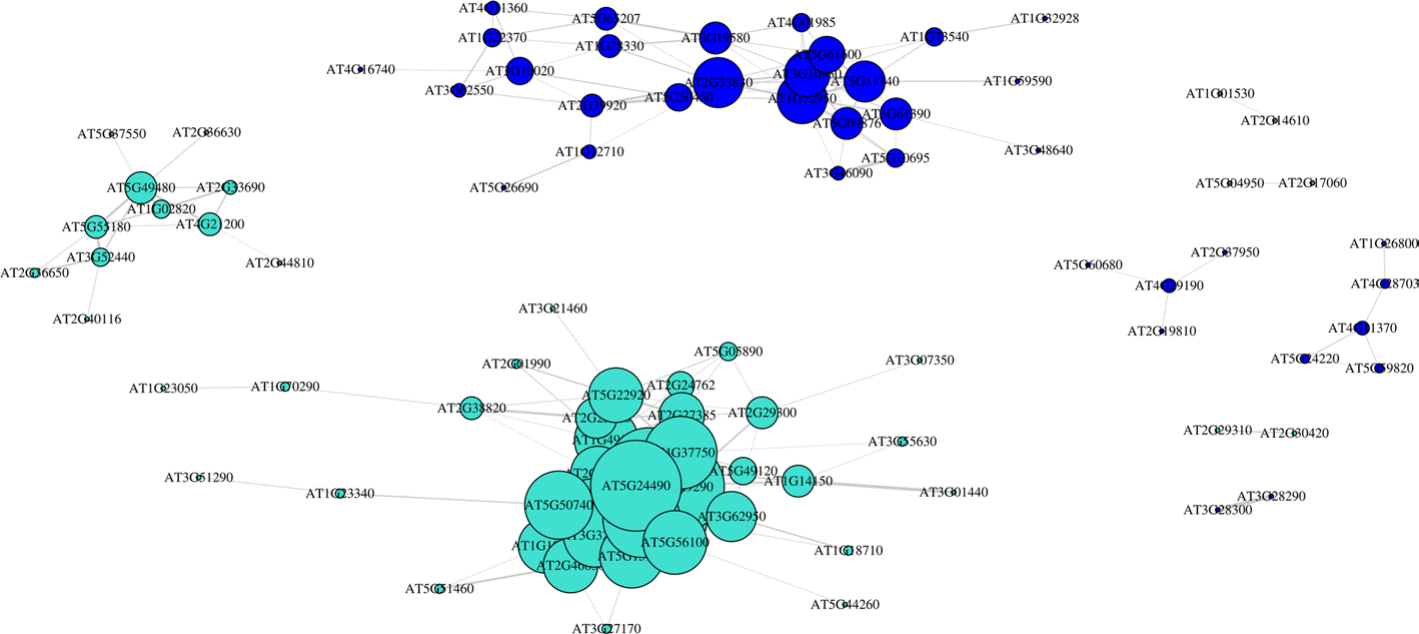
Gene correlation network for two WGCNA modules

A network analysis function we have implemented in *NetSeekR* builds network structures from the correlation of differentially expressed genes calculated in WGCNA. In Fig. 3 we show the network constructed for differentially expressed genes for two WGCNA modules (defined by the respective colors) with node sizes proportional to their degree and edge with proportional to correlation between genes.

**Fig. 4:**
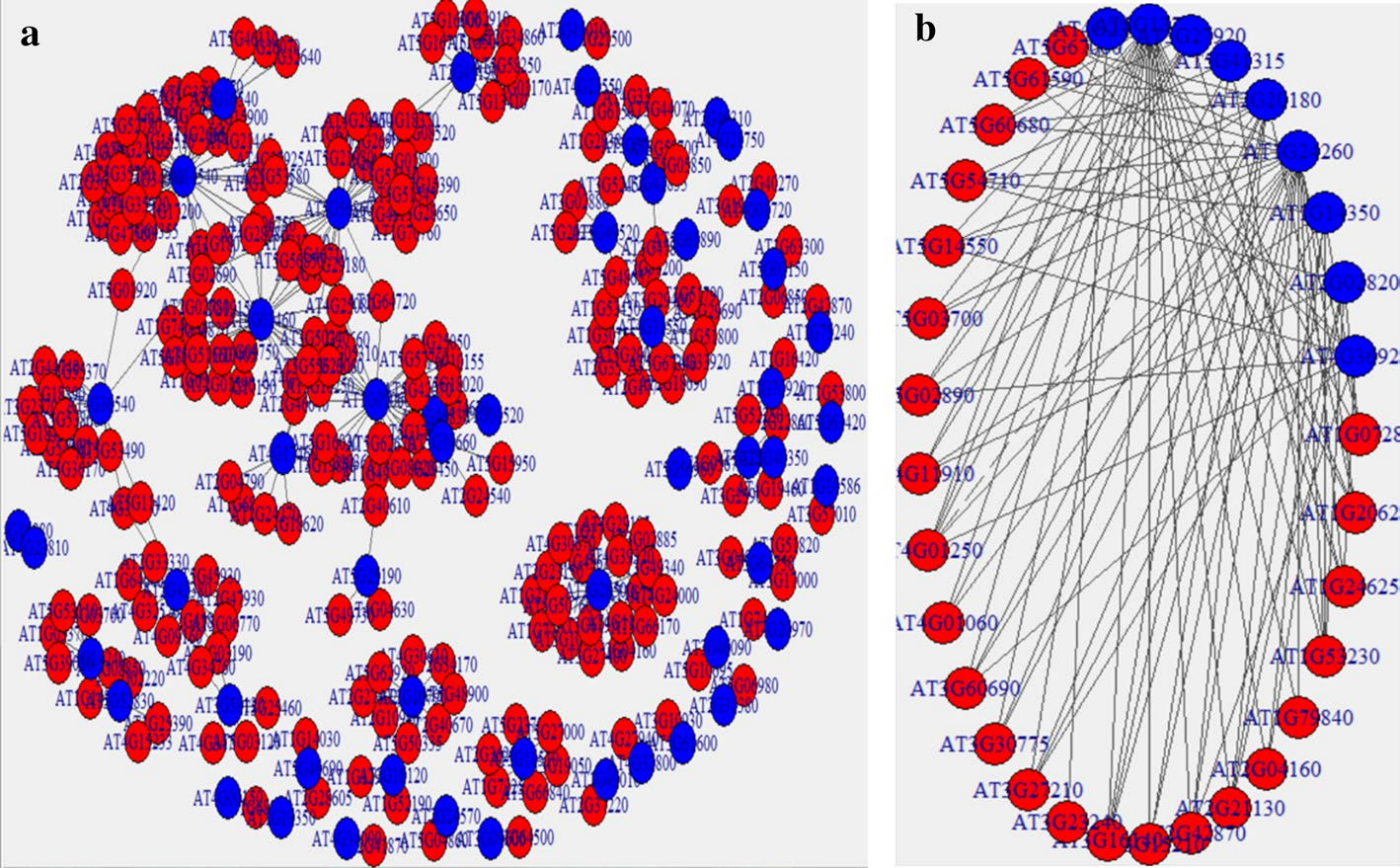
Regulatory networks of significant TF-gene interactions from **a** predicted from correlation analysis (WGCNA) and **b** inferred from DREM TF analysis. TFs are shown in blue and regulated genes in red; igraph’s Reingold–Tilford (**a**) and circular (**b**) layouts where used

Our package includes a regulatory network analysis function as well. In this function we are combining results from the WGCNA and DREM analysis to build regulatory graphs of significant Transcriptional Factor (TF)–gene interactions in the analyzed data. We take as inputs the modules obtained in the previous step in the WGCNA analysis and the lists of significant genes for each comparison set we use from the EdgeR/Sleuth output files. We identify the TFs in our dataset using the information from AtTFDB from the Arabidopsis Gene Regulatory Information Server (AGRIS) [28] database. AtTFDB contains information on approximately 1770 transcription factors (TFs) grouped into 50 families, based on the presence of conserved domains. We filter the significant genes interactions using a user-controlled threshold for the biweight midcorrelation calculated in WGCNA to generate a predicted regulatory network (Fig. 4A). We add to this the result of DREM analysis containing the list of regulatory TFs in our data. We convert this data into an incidence matrix which we then use to build a bipartite graph where one set is the list of TFs and the other the list of genes which those TFs are regulating (Fig. 4B).

In addition, the NetSeekR pipeline provides integration with several GO analysis R packages which can be used for assessing overexpression of differentially expressed genes, of clusters/modules of genes with correlated gene expression, or to perform GO analysis of other combinations of gene sets obtained from the pipeline.

## Conclusions

We have designed NetSeekR, a new integrated pipeline for large scale experimental designs that include RNA-Seq time series observations of gene expression dynamics of multiple treatments and multiple genotypes. The pipeline vertically integrates several reads mapping and analysis tools with regulatory and correlation network tools and provides additional network analysis, performance analysis and network visualization. Our methodology takes advantage of increasing availability of efficient data analysis pipelines to generate a flexible integration of genomics analysis tools from reads mapping to gene network analysis. This integration allows rapid design of gene expression analyses, easy comparison of several pipelines (using reads alignment and pseudo-alignment), differential gene expression analysis, network analysis, facilitating genomics discovery from large scale NGS data. The pipeline provides network prediction and analysis capabilities by integrating the inference of regulatory and correlation networks with network structure analysis and visualization tools. In this way the pipeline bridges the results of genomics data analysis to systems biology modeling and simulation.

### Availability and requirements

Project name: NetSeekR

Project home page: https://github.com/igbb-popescu-lab/NetSeekR

Docker image: https://hub.docker.com/r/af1065/netseekr

Operating system(s): Platform independent

Programming language: R

Other requirements: R package dependencies such as igraph, topGO, limma, dplyr, ggplot2, tidyr, tidygraph, Rgraphviz, biomaRT, edgeR, sleuth, and software packages DREM, STAR, Kallisto.

License: GNU GPL

Any restrictions to use by non-academics: none.

## Supplementary Information

**Additional file 1.** Brief description of functions implemented in NetSeekR.

**Additional file 2.** PDF file containing NetSeekR code and tutorial.

## Data Availability

Computer programs described in this work have been submitted in the additional files. The code is available at the project webpage: https://github.com/igbb-popescu-lab/NetSeekR. The example data described in this work is available at: https://doi.org/10.6084/m9.figshare.17447384.

## Abbreviations

RNA-Seq: RNA sequencing
DGE: Differential gene expression
WGCNA: Weighted Gene Correlation Network Analysis
DREM: Dynamic Regulatory Events Miner
log-FC: Log fold-change
Log-CPM: Log counts per million

## References

[1] Dobin A, Davis CA, Schlesinger F, Drenkow J, Zaleski C, Jha S, Batut P, Chaisson M, Gingeras TR (2013). STAR: ultrafast universal RNA-seq aligner. Bioinformatics.

[2] Pertea M, Kim D, Pertea GM, Leek JT, Salzberg SL (2016). Transcript-level expression analysis of RNA-seq experiments with HISAT, StringTie and Ballgown. Nat Protoc.

[3] Robinson MD, McCarthy DJ, Smyth GK (2010). edgeR: a Bioconductor package for differential expression analysis of digital gene expression data. Bioinformatics.

[4] Love MI, Huber W, Anders S (2014). Moderated estimation of fold change and dispersion for RNA-seq data with DESeq2. Genome Biol.

[5] Bray N, Pimentel H, Melsted P, Pachter L (2016). Near-optimal probabilistic RNA-Seq quantification. Nat Biotechnol.

[6] Patro R, Duggal G, Love MI, Irizarry RA, Kingsford C (2017). Salmon provides fast and bias-aware quantification of transcript expression. Nat Methods.

[7] Pimentel H, Bray NL, Puente S, Melsted P, Pachter L (2017). Differential analysis of RNA-seq incorporating quantification uncertainty. Nat Methods.

[8] Baruzzo G, Hayer KE, Kim EJ, Di Camillo B, FitzGerald GA, Grant GR (2017). Simulation-based comprehensive benchmarking of RNA-seq aligners. Nat Methods.

[9] Andrews S. FASTQC. A quality control tool for high throughput sequence data. https://www.bioinformatics.babraham.ac.uk/projects/fastqc/. Accessed 23 Dec 2021.

[10] Bolger AM, Lohse M, Usadel B (2014). Trimmomatic: a flexible trimmer for Illumina sequence data. Bioinformatics.

[11] Langfelder P, Horvath S (2008). WGCNA: an R package for weighted correlation network analysis. BMC Bioinform.

[12] Schulz MH, Devanny WE, Gitter A, Zhong S, Ernst J, Bar-Joseph Z (2012). DREM 2.0: Improved reconstruction of dynamic regulatory networks from time-series expression data. BMC Syst Biol.

[13] Csardi G, Nepusz T. The igraph software package for complex network research. InterJournal Complex Syst 2006;1695. http://igraph.org.

[14] Pedersen Thomas L. tidygraph: a tidy API for graph manipulation. https://cran.r-project.org/web/packages/tidygraph/index.html. Accessed 20 Oct 2020.

[15] Alexa A, Rahnenfuhrer J. topGO: topGO: enrichment analysis for gene ontology. https://bioconductor.riken.jp/packages/3.0/bioc/html/topGO.html. Accessed 20 Oct 2020.

[16] Müller K, Wickham H, Francois R, Bryan J. tibble: simple data frames. https://cran.r-project.org/web/packages/tibble/index.html. Accessed 20 Oct 2020.

[17] Durinck S, Spellman P, Birney E, Huber W (2009). Mapping identifiers for the integration of genomic datasets with the R/Bioconductor package biomaRt. Nat Protoc.

[18] Li H, Handsaker B, Wysoker A, Fennell T, Ruan J, Homer N, Marth G, Abecasis G, Durbin R (2009). 1000 Genome Project Data Processing Subgroup. The sequence alignment/map format and SAMtools. Bioinformatics.

[19] Liao Y, Smyth GK, Shi W (2014). featureCounts: an efficient general purpose program for assigning sequence reads to genomic features. Bioinformatics.

[20] Ritchie ME, Phipson B, Wu D, Hu Y, Law CW, Shi W, Smyth GK (2015). limma powers differential expression analyses for RNA-sequencing and microarray studies. Nucleic Acids Res.

[21] Cheng CY, Krishnakumar V, Chan AP, Thibaud-Nissen F, Schobel S, Town CD (2017). Araport11: a complete reannotation of the *Arabidopsis thaliana* reference genome. Plant J.

[22] Langfelder P, Miller JA, Horvath S. anRichment. Annotation and enrichment functions. https://horvath.genetics.ucla.edu/html/CoexpressionNetwork/GeneAnnotation/. Accessed 20 Oct 2020.

[23] Warde-Farley D, Donaldson SL, Comes O, Zuberi K, Badrawi R, Chao P, Franz M, Grouios C, Kazi F, Lopes CT (2010). The GeneMANIA prediction server: biological network integration for gene prioritization and predicting gene function. Nucleic Acids Res.

[24] Kerrien S, Aranda B, Breuza L, Bridge A, Broackes-Carter F, Chen C, Duesbury M, Dumousseau M, Feuermann M, Hinz U (2012). The IntAct molecular interaction database in 2012. Nucleic Acids Res.

[25] Szklarczyk D, Franceschini A, Kuhn M, Simonovic M, Roth A, Minguez P, Doerks T, Stark M, Muller J, Bork P (2011). The STRING database in 2011: functional interaction networks of proteins, globally integrated and scored. Nucleic Acids Res.

[26] Kanehisa M, Araki M, Goto S, Hattori M, Hirakawa M, Itoh M, Katayama T, Kawashima S, Okuda S, Tokimatsu T (2008). KEGG for linking genomes to life and the environment. Nucleic Acids Res.

[27] Conway JR, Lex A, Gehlenborg N (2017). UpSetR: an R package for the visualization of intersecting sets and their properties. Bioinformatics.

[28] Palaniswamy SK, James SHS, Lamb RS, Davuluri RV, Grotewold E (2006). AGRIS and AtRegNet. A platform to link cis-regulatory elements and transcription factors into regulatory networks. Plant Physiol.

