# Supplementary material for: NetSeekR: a network analysis pipeline for RNA-Seq time series data
Source: BMC Bioinformatics. 2022 Jan 28;23:54. doi: 10.1186/s12859-021-04554-1 (PMC8796424; doi:10.1186/s12859-021-04554-1)
Supplement: Supplementary file 2 — Additional file 2. PDF file containing NetSeekR code and tutorial. [file 12859_2021_4554_MOESM2_ESM.pdf]

NetSeekR

Set the working directory to the NetSeekR path.

```
#setwd(<<path/to/NetSeekR>>)
```

Unzip the NetSeekR file if necessary.

```
#unzip('NetSeekR.zip')
```

Load packages and source functions for NetSeekR.

Below is a template configuration file which needs to be edited per usage.

```
##
## ── Column specification ────────────────────────────────────────────────────────
## cols(
##   Argument_Type = col_character(),
##   Argument = col_character()
## )

## # A tibble: 46 x 2
##    Argument_Type              Argument
##    <chr>                      <chr>
##  1 analysis_type              transcriptional_effect_treatment_10_16_19
##  2 design_matrix              data/filenames_CA42AANXX_edit_conditions.csv
##  3 DREM                       data/software/drem2/
##  4 edger_adjustment_method    fdr
##  5 edger_lfc                  0.05
##  6 edger_method               separate
##  7 feature_counts_path        software/subread-1.6.5-Linux-x86_64/bin/featureCo…
##  8 kallisto                   TRUE
##  9 kallisto_bias              <NA>
## 10 kallisto_bootstrap_samples 10
## 11 kallisto_chromosomes       <NA>
## 12 kallisto_fasta_files       data/Araport11_genes.201606.cds.fasta.gz
## 13 kallisto_fastq_files       data/fastam/
## 14 kallisto_fr_stranded       <NA>
## 15 kallisto_fragment_length   <NA>
## 16 kallisto_fusion            <NA>
## 17 kallisto_genomebam         <NA>
## 18 kallisto_gtf               <NA>
## 19 kallisto_index             data/Araport11_genes.201606.cds.idx
## 20 kallisto_kmer_size         11
## 21 kallisto_make_unique       <NA>
```

```
## 22 kallisto_output_dir        data/Kallisto/
## 23 kallisto_path              kallisto
## 24 kallisto_plaintext         <NA>
## 25 kallisto_pseudobam         <NA>
## 26 kallisto_rf_stranded       <NA>
## 27 kallisto_sd                <NA>
## 28 kallisto_seed              <NA>
## 29 kallisto_single            <NA>
## 30 kallisto_single_overhang   <NA>
## 31 kallisto_threads           <NA>
## 32 sample_comparisons_file    data/treatment_comparisons.tsv
## 33 sample_covariates          genotype, condition, hour
## 34 significance_cutoff        0.05
## 35 sleuth_gene_mode           FALSE
## 36 sleuth_transcript_mode     TRUE
## 37 star                       TRUE
## 38 star_genomeDir             data/STAR/genome_Dir
## 39 star_genomeFastaFiles      data/Araport11_genes.201606.cds.fasta
## 40 star_path                  software/STAR
## 41 star_readFilesIn           data/fastam/
## 42 star_runMode               <NA>
## 43 star_runThreadN            12
## 44 star_sjdbGTFfile           data/Athaliana_447_Araport11.gene.gtf
## 45 star_sjdbOverhang          <NA>
## 46 tf_list                    data/Ath_TF_list.txt
```

Executing alignment with NetSeekR is completed with one function.

Ubuntu is required for the alignment function to conduct alignment since STAR and Kallisto are Linux tools.

```
# Save objects from the alignment function to a variable.
# The execute_script argument should be T (TRUE) on an Ubuntu machine
# if one wishes to execute the alignment tools.
alignment_results <- implement_alignment(arguments_file =
'data/transcriptional_effect_treatment_arguments.tsv', execute_script = F)
```

Differential gene expression software packages are executed next.

```
implement_differential_gene_expression(alignment_results)
```

Network analysis is then conducted assuming sets of differentially expressed genes are available.

Here, the edgeR results are analyzed as per the alignment_tool argument to the following function call:

```
# Network analysis for edgeR results.
```

```
implement_network_analysis(alignment_tool = 'edgeR', alignment_results =
alignment_results)
```

```
# Load all packages and source all functions.
if (!'pacman' %in% installed.packages()){
  install.packages('pacman')
}

library(pacman)


p_load(stringi, BiocManager, magrittr, readr, rlang, purrr, stringr,
ggplot2, devtools,
       flashClust, tidyr, networkD3, igraph, vroom, scales, reshape,
tibble, ggraph,
       tidygraph, dplyr, purrr)


# Install packages from Bioconductor.
bioconductor_packages <- c('limma', 'edgeR', 'topGO','WGCNA', 'biomaRt',
'Rgraphviz', 'STRINGdb', 'BiocManager')

for (package in bioconductor_packages){
  if (!package %in% installed.packages()){
    BiocManager::install(package, update = F)
  }
}

p_load(char = bioconductor_packages, install = F)

rm(bioconductor_packages)

BiocManager::install('pachterlab/sleuth')

# Prevent conflict of 'select' function in AnnotationDBi.
p_load(dplyr)

# Source all necessary scripts.
list.files('./scripts/', pattern = 'implement.*R$', full.names = TRUE)
%>%
  walk(source)
```

implement_alignment.R

```
# Function name: implement_alignment
# Purpose: Extract all arguments from the configuration file, decide
which
#          pipeline(s) to run, assemble arguments and implement each
#          decided pipeline.
# Input: Arguments file path.
# Output: Alignment decision data structure and processed configuration
file.
implement_alignment <- function(arguments_file, execute_script){

  # Convert arguments from configuration file
  # into a named list data structure.
  pipeline_input <- arguments_file %>%
    extract_pipeline_input_from_configuration()

  # Determine which pipeline(s) to execute,
  # and write directory trees for each tool
  # chosen.
  alignment_decision <- pipeline_input %>%
    decide_alignment_tool()

  # Extract paths to all written directories.
  alignment_directories <- alignment_decision %>%
    dplyr::select(directories) %>%
    unlist(use.names = FALSE)

  # Map over elements and create each directory.
  alignment_directories %>%
    map(dir.create,
        recursive = TRUE,
        showWarnings = FALSE)

  # Subset arguments from the configuration file
  # that are specific to an alignment tool.
  alignment_decision <- alignment_decision %>%

    mutate(
      arguments = map(key,
                      assemble_alignment_arguments,
                      pipeline_input
      )
    )

  # Set Boolean values for tools to be executed.
  Kallisto <- alignment_decision %>%
    align_tool_bool('key', 'kallisto')

  STAR <- alignment_decision %>%
    align_tool_bool('key', 'star')

  # Implement the selected alignment tool(s).

  # Kallisto
  if(Kallisto){
```

```
    # Subset arguments specific to Kallisto.
    alignment_decision %>%
      subset_tool_arguments('kallisto') %>%
      implement_kallisto(Kallisto_arguments = ., directories =
alignment_directories, execute_script)
  }

  # STAR
  if(STAR){

    # Subset arguments
    args <- alignment_decision %>%
      filter(str_detect(key, 'star')) %>%
      dplyr::select(arguments) %>%
      unnest(arguments)

    # Write bash scripts for STAR pipeline tools (STAR, Feature counts).
    star <- implement_STAR(STAR_arguments = args, directories =
alignment_directories, execute_script)

    # feature counts
    feature_counts_output <- star %>%
      str_replace('genome_Dir', 'Feature_counts')

    dir.create(feature_counts_output, showWarnings = FALSE)

    gtf <- pipeline_input %>%
      extract2('star_sjdbGTFfile')

    path_to_feature_counts <- pipeline_input %>%
      str_subset('.*bin/featureCounts.*')

    implement_feature_counts(output_dir = feature_counts_output,
annotation = gtf, feature_counts = path_to_feature_counts, genome_Dir =
star, execute_script)
  }
  return(list(alignment_decision, pipeline_input))
}




# Function name: extract_pipeline_input_from_configuration
# Purpose: Create a named list data structure for storing
#          and accessing arguments from the configuration file.
# Input: Arguments file path.
# Output: Arguments to pipeline in a named list.
extract_pipeline_input_from_configuration <- function(arguments_file){

  # Remove non-existant configurations.
  arg_file <- arguments_file %>%
    read_tsv(col_types = cols()) %>%
    filter(!(Argument_Type %>% is.na & Argument %>% is.na))
```

```
  # Extract argument types.
  arg_type <- arg_file %>%
    dplyr::select(Argument_Type) %>%
    unlist(use.names = F)

  # Link argument to argument type in a named list data structure.
  arg_file %>%
    dplyr::select(Argument) %>%
    unlist(use.names = F) %>%
    as.list() %>%
    set_names(arg_type)
}




# Function name: decide_alignment_tool
# Purpose: Write directory trees for decided tool.
# Input: processed configuration file (arguments list).
# Output: Tibble from which decisions on which downstream pipeline
#         decisions are to be made.
decide_alignment_tool <- function(pipeline_input){

  tibble(kallisto = pipeline_input %>% extract2('kallisto'),
         star = pipeline_input %>% extract2('star')
  ) %>%

    gather() %>%

    # Remove alignment tool which is not selected for implementing.
    filter(value == 'TRUE') %>%
    mutate(

      directories = map2(key, value,

                         # Determine which directory tree(s) to create.
                         ~dplyr::case_when(.x == 'kallisto' ~
pipeline_input %>%
                                     extract2('kallisto_output_dir') %>%
                                     write_kallisto_directory_tree,

                                   .x == 'star' ~ pipeline_input %>%
                                     extract2('star_genomeDir') %>%
                                     write_STAR_directory_tree
                         )
      )
    )
}




# Function name: write_kallisto_directory_tree
# Purpose: Create directory tree for Kallisto results.
```

```
# Input: Alignment pipeline-specific output directory.
# Output: Kallisto path list.
write_kallisto_directory_tree <- function(output_directory){

  # Attach output directories to the Kallisto directory created.
  Kallisto_paths <- tibble(kallisto = output_directory) %>%

    mutate(

      kallisto_quantifications = kallisto %>%
        str_replace(pattern = '$', replacement =
'Kallisto_quantifications/'
        ),

      kallisto_log_files = kallisto %>%
        str_replace(pattern = '$', replacement = 'Kallisto_log_files/')

    ) %>%
    gather() %>%
    dplyr::select(value) %>%
    list()
}




# Function name: write_STAR_directory_tree
# Purpose: Create directory tree for STAR results.
# Input: Alignment pipeline-specific output directory.
# Output: STAR path list.
write_STAR_directory_tree <- function(output_directory){

  # Save output directory basename to replace with pattern matching.
  STAR_node <- output_directory %>%
    basename()

  STAR_paths <- tibble(STAR = output_directory) %>%

    mutate(

      star_log_files = STAR %>%
        str_replace(pattern = STAR_node, replacement = 'STAR_log_files/'
        ),

      feature_counts = STAR %>%
        str_replace(pattern = STAR_node, replacement = 'Feature_counts/')

    ) %>%
    gather() %>%
    dplyr::select(value) %>%
    list()
}
```

```
# Function name: assemble_alignment_arguments
# Purpose: Subset arguments particular to an alignment tool
#          and ensure proper command formatting.
# Input: Alignment tool name and processed configuration file.
# Output: Formatted arguments for STAR or Kallisto in a tibble
#         data structure.
assemble_alignment_arguments <- function(alignment, pipeline_input){

  # These commands need an equals sign after the command.
  k_equals <-
'index|bootstrap_samples|seed|fragment_length|sd|threads|output_dir|kmer_
size' %>%
    str_replace_all(pattern = '\\|', replacement = '|kallisto_') %>%
    str_replace(pattern = '^', 'kallisto_') %>%
    str_split(pattern = '\\|') %>%
    unlist()

  # Subset arguments based on which tools they belong.
  arguments <- pipeline_input[grep(pattern = alignment, x =
names(pipeline_input))] %>%
    as_tibble() %>%
    gather() %>%
    drop_na() %>%
    filter(key != alignment) %>%

    # Place equal signs in command where appropriate.
    mutate_at(
      vars(key),
      list(
        ~(dplyr::if_else(condition = .x %>% is_in(k_equals),
                  true = .x %>% paste0('='),
                  false = .x)
        )
      )
    ) %>%
    mutate(
      # Reformat pipeline arguments to be usable by the alignment tool in
the command line.
      key = key %>%
        str_replace(pattern = paste0(alignment, '_'),
                    replacement = '--')
    )
}




# Function name: align_tool_bool
# Purpose: Check for tool existence in the decision data structure.
# Input: Alignment decision tibble, key with alignment tool name,
#        and a string for the alignment name.
# Output: Boolean value.
```

implement_alignment.R

```
align_tool_bool <- function(tib, column_name, str_to_detect){
  tib %>%
    dplyr::select(column_name) %>%
    unlist() %>%
    str_detect(str_to_detect) %>%
    any()
}




# Function name: subset_tool_arguments
# Purpose: Subset tool arguments from decision data structure
#          and pass them to tool-specific command processing functions.
# Input: Alignment decision data structure, tool name.
# Output: Tibble of arguments from selected tools.
subset_tool_arguments <- function(alignment_decision, tool_name){
  alignment_decision %>%
    filter(
      str_detect(key, !!tool_name)
    ) %>%
    dplyr::select(arguments) %>%
    unnest(cols = arguments)
}




# Function name: implement_kallisto
# Purpose: Write bash scripts for Kallisto index building and
quantification,
#          then execute them.
# Input: Arguments to Kallisto from configuration file, and all
directories
#        created.
implement_kallisto <- function(Kallisto_arguments, directories,
execute_script){

  # Separate indexing from quantifying arguments.
  index_arguments <- 'index|fasta|kmer|unique'

  organize_kallisto_arguments <- Kallisto_arguments %>%

    mutate(
      key = map_chr(key,
                    str_replace_all,
                    '_',
                    '-'
      ),
      index = map_lgl(key,
                      str_detect,
                      index_arguments
      ),
      quant = map_lgl(key,
```

```
                    str_detect,
                    index_arguments,
                    negate = TRUE
      )
    ) %>%

    # Mark arguments needed for both indexing and quantifying.
    mutate_at(
      vars(quant, index),
      list(
        ~dplyr::if_else(condition = str_detect(key, 'index'), true =
TRUE, false = .x)
      )
    ) %>%
    mutate_at(
      vars(index),
      list(
        ~dplyr::if_else(condition = str_detect(key, 'path'), true = TRUE,
false = .x)
      )
    )

  # Extract output directory for quantifications.
  output_dir <- organize_kallisto_arguments %>%
    filter(
      str_detect(key, 'output')
    ) %>%
    dplyr::select(value) %>%
    unlist(use.names = FALSE)

  # Extract output directory for log files.
  log_file_dir <- directories %>%
    str_subset('Kallisto_log_files')

  # Subset indexing-building arguments.
  arguments_to_build_index <- organize_kallisto_arguments %>%
    filter(index == TRUE) %>%
    dplyr::select(key, value)

  # Use fasta pattern to move fasta reference to end of command.
  fasta <- arguments_to_build_index %>%
    filter(
      str_detect(key, 'fasta-files')
    ) %>%
    dplyr::select(value) %>%
    unlist(use.names = FALSE)

  # Identify non-optional arguments.
  arg_core <- arguments_to_build_index %>%
    filter(
      str_detect(key, pattern = 'path|fasta|index')
    )
```

```
  # Identify optional arguments using already identified non-optional
arguments.
  extraneous_args <- anti_join(arguments_to_build_index, arg_core, by =
c('key', 'value')) %>%
    spread(key, value)

  # Assemble optional arguments.
  extraneous_args_type <- extraneous_args %>%
    colnames()

  extraneous_args_arg <- extraneous_args %>%
    unlist(use.names = FALSE)

  extraneous_args <- rbind(extraneous_args_type, extraneous_args_arg) %>%
    str_c(collapse = ' ') %>%
    str_remove(' TRUE')

  # Assemble non-optional arguments.
  arg_core <- arg_core %>%
    spread(key, value)

  arg_core_type <- arg_core %>%
    colnames()

  arg_core_arg <- arg_core %>%
    unlist(use.names = FALSE)

  # Assemble index-building shell script.
  build_index <- rbind(arg_core_type, arg_core_arg) %>%
    str_c(collapse = ' ') %>%

    # Move path to Kallisto the the start of the command.
    str_replace(pattern = '^',
                replacement = str_match(string = ., pattern = '--path.*')
%>% paste(' ', sep = '')
    ) %>%

    # Remove unnecessary arguments used as flags before.
    str_remove(pattern = ' --path.*'
    ) %>%

    str_replace('--fasta-files', replacement = str_match(string = .,
pattern = '--index.*'
    ) %>%
      str_replace(pattern = '= ', replacement = '=')
    ) %>%

    str_replace('--index=', 'index --index='
    ) %>%

    str_remove(' --index= .*'
    ) %>%

    paste(extraneous_args, collapse = ''
```

```
    ) %>%

    str_replace(pattern = '= ', replacement = '='
    ) %>%

    str_replace(pattern = '$', replacement = str_match(string = .,
pattern = fasta)
    ) %>%

    str_remove(pattern = str_match(string = ., pattern = paste0(' ' ,
fasta))
    ) %>%

    str_replace(pattern = fasta, replacement = paste0(' ', fasta)
    ) %>%

    str_replace('--kmer', ' --kmer'
    ) %>%

    paste('2>&1 | tee -a', paste0(log_file_dir, 'indexing.log')
    ) %>%

    str_remove('^--path ')

  # Write index building commands to file.
  indexing_script_location <- getwd() %>%
    paste0(., '/scripts/Kallisto_build_index.sh')

  indexing_script <- indexing_script_location %>%
    file()

  writeLines(build_index, indexing_script)

  close(indexing_script)

  FASTQ <- organize_kallisto_arguments %>%
    filter(
      str_detect(key, 'fastq')
    ) %>%
    dplyr::select(value) %>%
    unlist(use.names = FALSE)

  FASTQ_files <- tibble(forward = list.files(FASTQ, full.names = TRUE,
pattern = '*_1'),
                        reverse = list.files(FASTQ, full.names = TRUE,
pattern = '*_2')
  ) %>%
    mutate(
      sample = forward %>%
        basename %>%
        str_remove('_.*')
    )

  arguments_to_quantify_reads <- organize_kallisto_arguments %>%
```

```
    filter(quant == TRUE) %>%
    dplyr::select(key, value)

  arg_core <- arguments_to_quantify_reads %>%
    filter(
      str_detect(key, pattern = 'path|fastq|index|output')
    )

  extraneous_args <- anti_join(arguments_to_quantify_reads, arg_core, by
= c('key', 'value')) %>%
    spread(key, value)

  extraneous_args_type <- extraneous_args %>%
    colnames()

  extraneous_args_arg <- extraneous_args %>%
    unlist(use.names = FALSE)

  extraneous_args <- rbind(extraneous_args_type, extraneous_args_arg) %>%
    str_c(collapse = ' ') %>%
    str_replace_all('= ', '=')

  arg_core <- arg_core %>%
    spread(key, value)

  arg_core_type <- arg_core %>%
    colnames()

  arg_core_arg <- arg_core %>%
    unlist(use.names = FALSE)

  quant_paired_end_reads <- rbind(arg_core_type, arg_core_arg) %>%
str_c(collapse = ' ') %>% str_replace_all('= ', '=') %>%
    str_replace('^', replacement = str_match(string = ., pattern = '--
path.*') %>% paste(' ', sep = '')) %>%
    str_remove(' --path.*') %>%
    str_replace(pattern = '$', replacement = paste(' ', extraneous_args))
%>%
    str_remove('--path ') %>%
    str_remove('--fastq-files') %>%
    str_remove(FASTQ) %>%
    str_replace('$', paste(' ', FASTQ)) %>%
    str_replace_all(pattern = '[:space:]{2,}', replacement = ' ') %>%
    str_replace('--index', 'quant --index') %>%
    paste('2>&1 | tee ', log_file_dir %>% paste0('quant.log'))

  read_tib_to_save <- FASTQ_files %>%
    mutate(
      quantify_pe = quant_paired_end_reads,
      forard_and_reverse = map2(forward,
                                reverse,
                                paste
      ),
      quantify = pmap_chr(
```

```
        list(forward, reverse, sample, quantify_pe),
        ~str_replace(string = ..4, pattern = FASTQ, replacement = 
paste(..1, ..2, sep = ' ')
        ) %>%
          str_replace(string = ., pattern = output_dir, replacement = 
paste0(output_dir, 'Kallisto_quantifications/', ..3)) %>%
          str_replace(string = ., pattern = '$', replacement = ' &')
      )
    ) %>%
    dplyr::select(quantify) %>%
    # Place she-bang at top of file.
    add_row(quantify = '#!/bin/bash', .before = .1) %>%
    unlist(use.names = FALSE)

  quant_script_location <- output_dir %>%
    str_replace('data.*', 'scripts/') %>%
    paste0(., 'Kallisto_quantify.sh')

  quant_script <- quant_script_location %>%
    file()

  writeLines(read_tib_to_save, quant_script)

  close(quant_script)

  if (execute_script){
    system(indexing_script_location)
    system(quant_script_location)
  }

}




# Function name: implement_STAR
# Purpose: Write bash scripts for STAR index building and mapping,
#          then execute them.
# Input: Arguments to Kallisto from config file, and all directories
#        created.
implement_STAR <- function(STAR_arguments, directories, execute_script){

  index_arguments <- 
'fasta|runThread|genomeDir|genomeFastaFiles|sjdbGTFfile|sjdbOverhang'

  organize_STAR_arguments <- STAR_arguments %>%
    mutate(
      index = map_lgl(key,
                      str_detect,
                      index_arguments
      ),
      mapping = map_lgl(key,
                        str_detect,
```

```
                      index_arguments,
                      negate = TRUE
    )
  )

path <- organize_STAR_arguments %>%
  filter(
    str_detect(key, 'path')
  ) %>%
  dplyr::select(value) %>%
  unlist(use.names = FALSE)

genome_dir <- organize_STAR_arguments %>%
  filter(
    str_detect(key, 'genomeDir')
  ) %>%
  dplyr::select(key, value) %>%
  unlist(use.names = FALSE) %>%
  str_c(collapse = ' ')

output_dir <- directories %>%
  str_subset('STAR_log_files') %>%
  str_replace(pattern = 'data.*', 'scripts/')

runThread <- organize_STAR_arguments %>%
  filter(
    str_detect(key, 'runThread')
  ) %>%
  dplyr::select(key, value) %>%
  unlist(use.names = FALSE) %>%
  str_c(collapse = ' ')

reads <- STAR_arguments %>%
  filter(
    str_detect(key, 'readFilesIn')
  ) %>%
  dplyr::select(value) %>%
  unlist(use.names = FALSE) %>%
  list.files(full.names = T)

# Check if fastq files are zipped.
reads_zipped <- reads %>%
  first() %>%
  str_detect('.gz$')

read_tib <- tibble(
  forward = reads %>% str_subset(pattern = '_1'),
  reverse = reads %>% str_subset(pattern = '_2'),
  sample = map_chr(forward,
                   ~basename(.x) %>%
                     str_remove('_.*')
  )
)
```

```
  arguments_to_build_index <- organize_STAR_arguments %>%
    filter(index == TRUE) %>%
    dplyr::select(key, value) %>%
    spread(key, value)

  index_argument_type <- arguments_to_build_index %>%
    colnames()

  index_arguments <- arguments_to_build_index %>%
    unlist(use.names = FALSE)

  build_index_with <- rbind(index_argument_type, index_arguments) %>%
    str_c(collapse = ' ') %>%
    paste(path, '--runMode genomeGenerate', ., '2>&1 | tee -a ',
directories %>% str_subset('STAR_log_files/') %>%
paste0('STAR_index.log')) %>%
    str_replace(pattern = '$', replacement = ' &') %>%
    str_replace_all(pattern = '[:space:]{2,}', replacement = ' ')

  # Write bash script to build the index to a file in the scripts
directory.
  build_index_with <- paste('#!/bin/bash', build_index_with, sep = '\n')

  indexing_script <- paste0(output_dir, 'STAR_build_index.sh')

  indexing_script_file <- indexing_script %>%
    file()

  writeLines(build_index_with, indexing_script_file)

  close(indexing_script_file)

  FASTQ <- organize_STAR_arguments %>%
    filter(
      str_detect(key, 'readFilesIn')
    ) %>%
    dplyr::select(value) %>%
    unlist(use.names = FALSE)

  FASTQ_files <- FASTQ %>%
    list.files(full.names = TRUE) %>%
    str_c(collapse = ' ')

  arguments_to_map_reads <- organize_STAR_arguments %>%
    filter(mapping == TRUE) %>%
    dplyr::select(key, value) %>%
    spread(key, value)

  map_argument_type <- arguments_to_map_reads %>%
    colnames()

  map_arguments <- arguments_to_map_reads %>%
    unlist(use.names = FALSE)
```

```
  out_file_prefix <- directories %>%
    str_subset('genome_Dir')

  map_reads_with <- rbind(map_argument_type, map_arguments) %>%
    str_c(collapse = ' ') %>%
    str_remove('--path ') %>%
    paste(genome_dir) %>%
    paste(runThread)

  # Insert unzipping command if FASTQ files are zipped.
  if(reads_zipped){
    map_reads_with <- map_reads_with %>%
      paste('--readFilesCommand zcat')
  }

  map_reads_with <- map_reads_with %>%
    paste('--outFileNamePrefix', out_file_prefix) %>%
    paste(
      paste(' 2>&1 | tee -a ', directories %>%
              str_subset('STAR_log_files/') %>%
              paste0('STAR_mapping.log')
      )
    ) %>%
    str_replace_all(pattern = '[:space:]{2,}', replacement = ' ') %>%
    str_replace('$', ' &')

  read_tib_to_save <- read_tib %>%
    mutate(
      # Base command.
      map_reads_with = map_reads_with,

      # Combine forward with reverse read.
      forward_and_reverse = map2_chr(forward,
                                     reverse,
                                     paste
      ),

      # Write path to sample genome_dir for accessing sample-specific
mapped reads.
      sample_genome_dir = map_chr(sample,
                                  ~paste0(genome_dir %>% str_remove('^.*
'), '/', .x) %>%
                                    str_replace('//', '/')
      ),

      # Create sample genome directories for mapped reads.
      write_sample_genome_dir = map(sample_genome_dir,
                                    dir.create,
                                    recursive = TRUE,
                                    showWarnings = FALSE
      ),

      # Concatenate and string replace for writing full commands for
mapping with STAR.
```

```
      map_reads = pmap_chr(
        list(map_reads_with, forward_and_reverse, sample_genome_dir),
        ~str_replace(string = ..1, pattern = FASTQ, replacement = ..2)
%>%
          str_replace_all(string = ., pattern = genome_dir %>%
str_remove('^.* '), replacement = ..3 %>% paste0(., '/')) %>%
          str_replace(string = ., pattern = ..3, replacement = genome_dir
%>% str_remove('^.* ') %>% paste0(., '/')) %>%
          str_replace_all(string = ., pattern = '//', replacement = '/')
      )
    ) %>%
    dplyr::select(map_reads) %>%
    # Place shebang at top of file.
    add_row(map_reads = '#!/bin/bash', .before = 1) %>%
    unlist(use.names = FALSE)

  mapping_script_location <- paste0(output_dir %>% str_replace('data.*',
'scripts/'), 'STAR_map_reads.sh')

  mapping_script <- mapping_script_location %>%
    file()

  writeLines(read_tib_to_save, mapping_script)

  close(mapping_script)

  if (execute_script){
    system(indexing_script)
    system(mapping_script_location)
  }



  return(out_file_prefix)
}





# Function name: implement_feature_counts
# Purpose: Write bash script for feature counts to execute.
# Input: path to feature counts output, gtf, genome directory containing
sam files,
#        and path to the feature counts program.
implement_feature_counts <- function(output_dir, annotation, genome_Dir,
feature_counts, execute_script){

  feature_counts_command <- paste(feature_counts, '-a', annotation, '-o
x') %>%
    str_replace('x$', paste0(output_dir, 'clean_counts.txt'))

  # Format feature counts arguments using samples from STAR output.
  feature_counts_commands <- genome_Dir %>%
```

```
    list.files(recursive = T, full.names = TRUE) %>%
    tibble(sam_output = .) %>%
    mutate(

      sample = map_chr(sam_output,
                       ~dirname(.x) %>%
                         basename()
      ),

      feature_counts = feature_counts_command,

      fc = map2_chr(feature_counts,
                    sample,
                    ~str_replace(string = .x,
                                 pattern = 'clean_counts.txt',
                                 replacement = paste(.y,
'clean_counts.tsv', sep = '_')
                    )
      ),

      fc_output = map2_chr(fc,
                           sam_output,
                           ~paste(.x, .y)
      )
    ) %>%
    dplyr::select(fc_output) %>%
    add_row(fc_output = '#!/bin/bash', .before = 1) %>%
    unlist(use.names = FALSE)

  feature_counts_script_location <- genome_Dir %>%
    str_replace('data.*', 'scripts/') %>%
    paste0(., 'feature_counts.sh')

  feature_counts_script <- feature_counts_script_location %>%
    file()

  writeLines(feature_counts_commands, feature_counts_script)

  close(feature_counts_script)

  if (execute_script){
    system(feature_counts_script_location)
  }
}
```

implement_differential_gene_expression.R

```
# Function name: implement_differential_gene_expression
# Purpose: Extract alignment function output and execute differential
gene
#          expression testing depending on the upstream alignment or
pseudo-alignment
#          software used.
# Input: Alignment function results.
# Output: Alignment function results and processed configuration file.
implement_differential_gene_expression <- function(alignment_results){

  # Separate alignment results (tibble with tool commands
  # and directories) from the pipeline input (configuration
  # file exraction).
  alignment <- alignment_results %>%
    dplyr::first()

  pipeline_input <- alignment_results %>%
    dplyr::last()

  # Load experimental design matrix file.
  design_matrix <- pipeline_input %>%
    extract2('design_matrix') %>%
    read_csv(col_types = cols())

  # Separate covariates for column selection.
  sample_covariates <- pipeline_input %>%
    extract2('sample_covariates') %>%
    split_and_unlist_conditions()

  # Map design matrix contents with sets of samples to
  # compare in differential expression testing.
  sample_comparisons <- pipeline_input %>%
    extract2('sample_comparisons_file') %>%
    read_tsv(col_names = FALSE, col_types = cols()) %>%
    extract_sample_comparison_sets(design_matrix, sample_covariates)

  kallisto <- alignment %>%
    extract2('key') %>%
    str_detect('kallisto') %>%
    any()

  STAR <- alignment %>%
    extract2('key') %>%
    str_detect('star') %>%
    any()

  ## Sleuth non-functional.
  if(kallisto){

    #readline(prompt="Enter name: ")
    implement_sleuth(alignment, pipeline_input, design_matrix,
sample_comparisons, sample_covariates)
  }
```

```
  if(STAR){
    implement_edgeR(alignment, pipeline_input, design_matrix,
sample_comparisons)
  }

}




# Function name: split_and_unlist_conditions
# Purpose: Separate covariates from list of conditions.
# Input: Condition names from experimental design matrix and
#        configuration file.
# Output: Split list with spaces removed.
split_and_unlist_conditions <- function(covariate_character_vector){
  covariate_character_vector %>%
    str_split(pattern = ',') %>%
    unlist(use.names = FALSE) %>%
    str_remove('[:space:]')
}





# Function name: extract_sample_comparison_sets
# Purpose: Associate sample sets with sample comparisons.
# Input: Sample comparisons file, experimental design matrix,
#        and the split sample conditions.
# Output: Comparison sets associated with file names.
extract_sample_comparison_sets <- function(comparison_sets,
design_matrix, sample_conditions){
  comparison_sets %>%

    # Combine sample identifiers in sample comparisons file with
    # a pipe for searching the experimental design matrix with.
    unite(col = samples,
          sep = '|') %>%

    dplyr::transmute(
      comparison_set = map(samples,
                           ~dplyr::filter(design_matrix,
                                   stringr::str_detect(!!sym('sample'),
.x)
                           )
      ),

      file_name = purrr::map_chr(comparison_set,

~write_differential_testing_file_name(comparison_set = .x,

sample_conditions)
      )
    )
}
```

```
# Function name: write_differential_testing_file_name
# Purpose: Combine conditions from each sample comparison
#          set to create a file name with.
# Input: Comparison set, and split conditions.
# Output: A file name with '_vs_' between conditions compared.
write_differential_testing_file_name <- function(comparison_set,
sample_conditions){
  comparison_set %>%
    dplyr::select(condition) %>%
    unique() %>%
    arrange(desc(condition)) %>%
    unlist(use.names = FALSE) %>%
    str_c(collapse = '_vs_') %>%
    str_replace_all(pattern = ' ', replacement = '_')
}
```

```
# Function name: implement_sleuth
# Purpose: Run Sleuth on one sample comparison set at a time.
# Input: Alignment results data structure, processed configuration file,
#        experimental design matrix, sample comparison sets, and split
#        sample comparisons.
implement_sleuth <- function(alignment, pipeline_input, design_matrix,
sample_comparisons, sample_covariates){

  # Provide access to Kallisto directories.
  kallisto_directories <- alignment %>%
    filter(key == 'kallisto') %>%
    dplyr::select(directories) %>%
    unlist(use.names = F)

  # List sample quantification results.
  quantification_files <- kallisto_directories %>%
    str_subset('Kallisto_quantifications') %>%
    list.files(full.names = TRUE) %>%
    tibble(path = .) %>%
    mutate(
      # Ensure no double forward-slash.
      path = map_chr(path,
                     str_replace,
                     '//',
                     '/'),
      # Give sample name.
      sample = map_chr(path,
                       str_remove,
                       '.*/'
      )
    )
```

```
  # Reset quantification file paths in design matrix and comparison sets
  # from the original design matrix to the sample quantification results.
  design_matrix <- design_matrix %>%
    mutate(
      path = quantification_files %>%
        dplyr::select(path) %>%
        unlist(use.names = F)
    )

  #
  sample_comparisons <- sample_comparisons %>%
    unnest(cols = comparison_set) %>%
    left_join(quantification_files, by = 'sample') %>%
    dplyr::select(-path.x) %>%
    dplyr::rename(path = path.y) %>%
    drop_na() %>%
    nest(comparison_set = -file_name) %>%
    #nest(data = c(sample, genotype, condition, hour, which_replicate,
testing_condition, path)) %>%
    mutate(
      testing_condition = map(comparison_set,
                              structure_covariates_for_DGE_testing,
                              sample_covariates
      ),
      comparison_set = map(comparison_set,
                           separate_conditions,
                           sample_covariates
      )
    )

  # Determine which mode(s) to execute Sleuth in.
  gene_mode <- pipeline_input %>%
    extract2('sleuth_gene_mode')

  transcript_mode <- pipeline_input %>%
    extract2('sleuth_transcript_mode')

  sleuth_mode <- gene_mode %>%
    select_sleuth_mode(transcript_mode)

  # Place Sleuth results next to Kallisto.
  sleuth_path <- kallisto_directories %>%
    str_subset(pattern = 'Kallisto/$') %>%
    str_replace('Kallisto', 'Sleuth')

  transcript_level <- str_detect(sleuth_mode, 'transcript')

  gene_level <- str_detect(sleuth_mode, 'gene')

  transcript_and_gene_level <- str_detect(sleuth_mode,
'transcript_and_gene_level')

  analysis_type <- pipeline_input %>%
```

```
    extract2('analysis_type')

  sleuth_level_directories <- tibble(sleuth_path, transcript_level,
gene_level, transcript_and_gene_level) %>%
    mutate(
      transcript_level = case_when(transcript_level ~ paste0(sleuth_path,
analysis_type, '/transcript_level/')
      ),
      gene_level = case_when(gene_level ~ paste0(sleuth_path,
analysis_type, '/gene_level/')
      )
    ) %>%
    gather() %>%
    filter(
      str_detect(value, paste0('Sleuth/', analysis_type)
      )
    ) %>%
    mutate(
      write_directory = map(value,
                            dir.create,
                            recursive = TRUE,
                            showWarnings = FALSE
      )
    ) %>%
    dplyr::select(-write_directory)

  if(transcript_and_gene_level | gene_level){
    target_mapping <- design_matrix %>%
      slice(1) %>%
      gene_level_analysis_data_structure() %>%
      dplyr::select(ttg) %>%
      unnest()
  }

  sample_comparisons <- sample_comparisons %>%
    mutate(
      testing_condition_formula = map(testing_condition,
                                      extract_single_testing_condition
      )
    )

  if(transcript_and_gene_level){
    transcript_comparisons <- sample_comparisons %>%
      transcript_prep()

    gene_comparisons <- sample_comparisons %>%
      gene_prep(target_mapping)

    comparisons <- bind_rows(transcript_comparisons, gene_comparisons)
  }

  if(gene_level & !(transcript_and_gene_level | transcript_level)){
    comparisons <- sample_comparisons %>%
      gene_prep(target_mapping)
```

```
  }

  if(transcript_level & !(gene_level | transcript_and_gene_level)){
    comparisons <- sample_comparisons %>%
      transcript_prep()
  }


  rm(sample_comparisons)

  comparisons <- comparisons %>%
    left_join(sleuth_level_directories) %>%
    dplyr::rename(level_directory = value) %>%
    dplyr::select(-key)

  comparisons <- comparisons %>%
    mutate(
      pca = map2(prep,
                 testing_condition_formula,
                 run_sleuth_pca
      ),
      pca_save = pmap(
        list(pca, level_directory, file_name),
        save_sleuth_pca
      ),
      fit = map2(prep,
                 testing_condition_formula,
                 run_sleuth_fit
      ),
      lrt = map(fit,
                run_sleuth_lrt
      ),
      lrt_results = map(lrt,
                        extract_sleuth_lrt_results
      ),
      save_lrt_results = pmap(
        list(lrt_results, level_directory, file_name),
        save_sleuth_results
      )
    )
}




# Function name: structure_covariates_for_DGE_testing
# Purpose: Search for conditions in the comparison sets which vary in a
column.
# Input: Comparison set and split sample conditions.
# Output: Unique key, value pairs for conditions.
structure_covariates_for_DGE_testing <- function(comparison_set,
sample_conditions){
  condition_count <- sample_conditions %>%
    length()
```

```
  comparison_set <- comparison_set %>%
    extract(col = condition,
            into = sample_conditions,
            regex = rep('(.*)', times = condition_count) %>%
              paste(collapse = ' ')
    ) %>%
    dplyr::select(
      all_of(sample_conditions)
    ) %>%
    distinct()

  row_num <- comparison_set %>%
    nrow()

  comparison_set %>%
    gather() %>%
    group_by(key) %>%
    mutate(
      variant = value %>% n_distinct
    ) %>%
    filter(variant > 1) %>%
    mutate(
      test = map2_chr(key,
                      value,
                      paste0)
    ) %>%
    dplyr::select(key,
           test
    ) %>%
    distinct()
}



# Function name: separate_conditions
# Purpose: Separate condition column into multiple columns  based on the
covariates.
# Input: Sample comparison set and split condition list.
# Output: A sample comparison set with one column for each covariate.
separate_conditions <- function(clean_design_matrix, conditions){
  condition_count <- conditions %>%
    length()

  clean_design_matrix %>%
    tidyr::extract(col = condition,
                   into = conditions,
                   regex = rep('(.*)', times = condition_count) %>%
                     paste(collapse = ' ')
    )
}
```

```
# Function name: select_sleuth_mode
# Purpose: Determine  at what level to run Sleuth: gene, transcript, or
both.
# Input: Boolean values for gene mode and transcript mode.
# Output: A mode to run Sleuth in.
select_sleuth_mode <- function(gene_mode, transcript_mode){
  if_else(condition = (gene_mode == 'TRUE'),
          true = if_else(condition = (transcript_mode == 'TRUE'),
                         true = 'transcript_and_gene_level',
                         false = 'gene_level'),
          false = if_else(condition = (transcript_mode == 'TRUE'),
                          true = 'transcript_level',
                          false = 'NULL')
  )
}




# Function name: gene_level_analysis_data_structure
# Purpose: Removes splicing notation from one quantification file result
to use for gene level Sleuth analysis.
# Input: Experimental design matrix.
# Output: Gene to transcript mapping.
gene_level_analysis_data_structure <- function(design_matrix){
  design_matrix <- design_matrix %>%
    mutate(
      path = map_chr(path,
                     list.files,
                     '^abundance.tsv$',
                     full.names = TRUE,
                     recursive = T
      ),
      reads = map(path,
                  read_tsv,
                  col_types = cols())
    ) %>%
    unnest()

  # Check if the gene names have been extracted from
  # transcripts for the first quantification dataset.
  gene_column_existent <- design_matrix %>%
    colnames() %>%
    str_detect('gene') %>%
    any()

  if(!gene_column_existent){
    design_matrix <- design_matrix %>%
      group_by(target_id) %>%
      nest() %>%
      mutate(
        gene = target_id %>%
          str_remove('[[:punct:]].*')
```

```
      ) %>%
      unnest()

  } else{
    design_matrix <- design_matrix
  }

  design_matrix %>%
    group_by(sample,
             path,
             condition,
             which_replicate
    ) %>%
    nest() %>%
    mutate(
      data = map2(data,
                  path,
                  write_tsv
      ),
      ttg = map(data,
                dplyr::select,
                gene,
                target_id
      ),
      path = map_chr(path,
                     dirname)
    ) %>%
    dplyr::select(-data)
}



# Function name: extract_single_testing_condition
# Purpose: Select a single covariate to test with by
#          identifying the condition column with varying
#          contents across samples.
# Input: Split sample conditions.
# Output: Covariate which varies in its contents.
extract_single_testing_condition <- function(sample_conditions){

  count_comparisons <- sample_conditions %>%
    dplyr::select(key) %>%
    plyr::count()

  key_no_variance <- count_comparisons %>%
    filter(freq < 2) %>%
    dplyr::select(key)

  if(nrow(key_no_variance) >=1 ){
    no_variance_warning <- key_no_variance %>%
      unlist(use.names = FALSE) %>%
      str_c(collapse = ' and ') %>%
```

```
      paste0('Warning: Removing ', ., '. Sleuth needs two comparisons in
each category.')

    message(no_variance_warning)
  }

  sample_conditions %>%
    anti_join(key_no_variance, by = 'key') %>%
    ungroup() %>%
    dplyr::select(key) %>%
    unlist(use.names = FALSE) %>%
    paste0('~', .) %>%
    unique()
}




# Function name: transcript_prep
# Purpose: A Sleuth_prep implementation specific
#          to transcript analysis.
# Input: A sample comparison set.
# Output: Sleuth prep object.
transcript_prep <- function(comparisons){
  comparisons %>%
    mutate(
      prep = map2(comparison_set,
                  testing_condition_formula,
                  ~sleuth::sleuth_prep(sample_to_covariates = .x,
                                       full_model = .y %>%
                                         unique() %>%
                                         as.formula(),
                                       gene_mode = FALSE)
      ),
      key = 'transcript_level'
    )
}





# Function name: gene_prep
# Purpose: A Sleuth_prep implementation specific to gene analaysis.
# Input: A sample comparison set.
# Output: Sleuth prep object.
gene_prep <- function(comparisons, mapped_targets){
  comparisons %>%
    mutate(
      testing_condition_formula = map(testing_condition_formula,
                                      as.formula
      ),
      prep = map2(comparison_set,
                  testing_condition_formula,
```

```
                    sleuth::sleuth_prep,
                    target_mapping = mapped_targets,
                    aggregation_column = 'gene',
                    extra_bootstrap = TRUE,
                    gene_mode = TRUE
            ),
            key = 'gene_level'
        )
}




# Function name: run_sleuth_pca
# Purpose: Plot PCA plots for a comparison.
# Input: Sleuth prep object and a covariate to
#        color with.
# Output: PCA plot for comparison.
run_sleuth_pca <- function(prep, covariate_color){
  covariate_color <- covariate_color %>%
    str_remove('^~')

  prep %>%
    sleuth::plot_pca(color_by = covariate_color, text_labels = FALSE)
}




# Function name: save_sleuth_pca
# Purpose: Save PCA plots for a comparison.
# Input: PCA plot to save, Parent directory, and file name.
# Output: ggsave object.
save_sleuth_pca <- function(pca_plot, level_path, file_name){
  level_path <- level_path %>%
    basename() %>%
    paste0(level_path, ., '_PCA')

  dir.create(level_path, showWarnings = FALSE, recursive = TRUE)

  pca_plot %>%
    ggsave(filename = paste0(file_name, '.pdf'), path = level_path,
device = 'pdf')
}




# Function name: run_sleuth_fit
# Purpose: Measurement error model fitting with Sleuth.
# Input: Sleuth prep object and testing formula.
# Output: Sleuth fit object.
run_sleuth_fit <- function(prep, formula_string){
  prep %>%
```

```
    sleuth::sleuth_fit(obj = .,
                       formula = formula_string %>%
                         as.formula,
                       'full'
    ) %>%
    sleuth::sleuth_fit(obj = .,
                       formula = ~1,
                       'reduced')
}




# Function name: run_sleuth_lrt
# Purpose: Perform Likelihood Ratio Test.
# Input: Sleuth fit object.
# Output: LRT results.
run_sleuth_lrt <- function(fit){
  fit %>%
    sleuth::sleuth_lrt(obj = .,
                       'reduced',
                       'full')
}




# Function name: extract_sleuth_lrt_results
# Purpose: Extract Likelihood Ratio test results from a sleuth object.
# Input: Sleuth object
# Output: Likelihood ratio test results.
extract_sleuth_lrt_results <- function(lrt){
  lrt %>%
    sleuth::sleuth_results(test = 'reduced:full',
                           test_type = 'lrt')
}




# Function name: save_sleuth_results
# Purpose: Save LRT results to a file for a comparison.
# Input: LRT results, analysis level, file name.
# Output: None.
save_sleuth_results <- function(results, level_path, file_name){
  level_path <- level_path %>%
    basename() %>%
    paste0(level_path, ., '_results/')
  dir.create(level_path, showWarnings = FALSE, recursive = TRUE)

  results %>%
    write_tsv(path = paste0(level_path, file_name, '.tsv'))
}
```

```
# Function name: implement_edgeR
# Purpose: Conduct differential testing on STAR
#          mapped reads with edgeR.
# Input: Alignment decision data structure, processed configuration
#        file, experimental design matrix, and sample comparison sets.
implement_edgeR <- function(alignment, pipeline_input, design_matrix,
sample_comparisons){
  STAR_counts_path <- alignment %>%
    filter(key == 'star') %>%
    dplyr::select(directories) %>%
    unlist(use.names = FALSE) %>%
    str_subset(pattern = 'Feature_counts') %>%
    list.files(full.names = TRUE)

  design_matrix <- design_matrix %>%
    mutate(
      path = STAR_counts_path
    )

  no_return <- STAR_counts_path %>%
    tibble(files = .) %>%
    mutate(
      # Select the column one and column two from feature counts data; to
be ran with submitted data.
      counts_data = map(files, ~read_tsv(.x, col_names = TRUE, skip = 1,
col_types = cols()) %>% dplyr::select(1, 2)
      )
    ) %>%
    filter_counts_data(design_matrix = design_matrix) %>%
    edgeR_preliminary(count_keeps = ., samplename = design_matrix,
edgeR_directory = STAR_counts_path, comparisons = sample_comparisons,
pipeline_input)
}




# Function name: filter_counts_data
# Purpose: filter the lowly expressed genes for edgeR analysis.
# Input: counts, experimental design matrix.
# Output: data frame of counts for each sample.
filter_counts_data <- function(counts, design_matrix){
  data <- data.frame(counts$counts_data)

  # storing data as data frame
  total_samples <- nrow(counts)*2
  ans <- seq(2,total_samples,2)
  data <- data[c(1, ans)]

  # Load the sample information and include the header.
  samplename <- design_matrix
```

```
  #(optional) naming the samples
  #d=colnames(sleuth_table)[1]
  d="target_id"
  x <- samplename$`sample`
  a <- c(d,x)
  colnames(data) <- a
  rowname<-data[c(1)]

  #filterng the data
  keep <- rowSums(cpm(data[,2:length(data)]) > 0.5) >= 2
  data[keep,]
}




# Function name: edgeR_preliminary
# Purpose: process input data for Edge R analysis followed by the
analysis and set the directory where data has to be saved
# Input: kept counts, a sample name, edgeR directory,
#        comparison matrix, and the extracted configuration
#        data structure.
edgeR_preliminary <- function(count_keeps, samplename, edgeR_directory,
comparisons, pipeline_input){

  edgeR_output_directory <- edgeR_directory %>%
    dplyr::first() %>%
    str_replace(pattern = 'STAR.*', replacement = 'edgeR/') %>%
    paste0(pipeline_input$analysis_type, '/')

  dir.create(edgeR_output_directory, recursive = TRUE, showWarnings =
FALSE)

  # grouping the samples
  edgeR_comparision_set1 <- gsub(" ","_",samplename$condition)
  group <- factor(edgeR_comparision_set1)
  y <-
DGEList(count_keeps[,2:length(count_keeps)],group=group,genes=count_keeps
[c(1)])
  logcounts <- cpm(y,log=TRUE)
  y <- calcNormFactors(y, method='TMM')
  logcounts <- cpm(y,log=TRUE)
  #########################################################
  # forming the design matrix from DGE list generated before
  design.mat <- model.matrix(~ 0 + y$samples$group)

  colnames(design.mat) <- levels(y$samples$group)
  v <- voom(y,design.mat)
  #fitting the model
  vfit <- lmFit(v, design.mat)
  ###############contrast matrix1#########################################
  #can be of any choice
  comparisons$file_name %>%
```

```
    tibble(C = .) %>%
    mutate(
      B = map_chr(C, ~str_remove(string = .x, pattern = '_vs.*')),
      A = map_chr(C, ~str_remove(string = .x, pattern = '.*vs_')),
      all = pmap(list(C, A, B), ~paste0(..1, ' = ', ..2, ' - ', ..3))
    ) %>%
    mutate(
      make_contrasts = map(all, ~makeContrasts(contrasts = .x, levels =
group)),
      vfit = map(make_contrasts, ~contrasts.fit(fit = vfit, contrasts =
.x)),
      tfit = map(vfit, ~treat(fit = .x, lfc = pipeline_input$edger_lfc
%>% as.numeric())),
      dt = map(tfit, ~decideTests(object = .x, adjust.method =
pipeline_input$edger_adjustment_method, p.value =
pipeline_input$significance_cutoff)),
      top_tables = map2(tfit, all, ~topTable(fit = .x, coef = .y, sort.by
= 'p', n = 'Inf')),
      write_out = map2(top_tables, C, ~write_tsv(x = .x, path =
paste0(edgeR_output_directory, .y, '.tsv')))
    )
}
```

```
# Function name: implement_network_analysis
# Purpose: Performs network visualization and find hubbed genes
#          in the network using Go enrichment analysis also.
# Input: A string specifying which differential gene expression
#        was used upstream.
# Output: Network visualization and GO terms associated with
#         significant genes.
implement_network_analysis <- function(alignment_tool, alignment_results,
exectute){

  alignment_decision <- alignment_results %>%
    dplyr::first()
  pipeline_input <- alignment_results %>%
    dplyr::last()

  # WGCNA
  if(alignment_tool %>% str_detect('kallisto|KALLISTO|Kallisto')){
    wgcna_input <- alignment_decision %>%
      filter(key == 'kallisto') %>%
      dplyr::select(directories) %>%
      unlist(use.names = FALSE) %>%
      first() %>%
      str_replace('Kallisto', paste0('Sleuth/', pipeline_input %>%
extract2('analysis_type'), '/gene_level/gene_level_results'))
  }

  if(alignment_tool %>% str_detect('STAR|star|Star')){
    wgcna_input <- pipeline_input %>%
      extract2('star_genomeDir') %>%
      str_replace('STAR.*', paste0('edgeR/',
pipeline_input$analysis_type, '/')) %>%
      str_replace(pattern = '//', replacement = '/')


    # Remove tags after gene names in edgeR results.
    deg_files <- wgcna_input %>%
      list.files(path = ., pattern = '.tsv$', full.names = TRUE,
recursive = FALSE) %>%
      tibble(deg_file = .)

    test_for_extraneous <- deg_files %>%
      use_series(deg_file) %>%
      dplyr::first() %>%
      read_tsv(col_types = cols()) %>%
      use_series(target_id) %>%
      dplyr::first() %>%
      unlist(use.names = FALSE)

    # str matching specific to test data.
    if(test_for_extraneous %>% str_detect('\\..*')){
      deg_files_remove <- deg_files %>%
        mutate(
          deg_data = map(deg_file,
                         ~read_tsv(.x, col_types = cols()) %>%
```

```
                            mutate(
                              target_id = map_chr(target_id,
                                                  ~gsub(pattern = '\\..*',
replacement = '', x = .x)
                              )
                            )
          ),
          save_deg_data = map2(deg_data,
                               deg_file,
                               write_tsv
          )
        )
    }
  }

  message('Running WGCNA.')

  expr2 <- wgcna_input_data(wgcna_input, pipeline_input)

  significant_hits <- expr2 %>%
    dplyr::select(original_filter) %>%
    unnest(cols = original_filter) %>%
    distinct()

  expr2 <- expr2 %>%
    wgcna_plot_sample_tree() %>%
    wgcna_plot_power_results() %>%
    wgcna_plot_power_histogram() %>%
    wgcna_clustering()


  message('Performing GO enrichment.')
  GO_results <- implement_GO_enrichment(deg_tool = wgcna_input,
alignment_results = alignment_results) %>%
    post_process_GO_results()

  expr2 <- expr2 %>%
    mutate(
      GO_results = GO_results
    )

  # DREM
  message('Writing executable files for DREM.')
  time_series_count_data <- DREM_main(pipeline_input = pipeline_input,
wgcna_input = wgcna_input, significant_hits, execute)
  # Overlap differentially expressed genes with network
  DREM_network_overlap(pipeline_input, deg_files)

  message('Conducting network analysis.')
  network_analysis_results <- mapping_network_analysis(expr2)

  adjacency_matrices <- expr2 %>%
    mutate(
      comparison_adj_mat = map(clustering_res, ~.x$adjacency)
```

```
    ) %>%
    dplyr::select(comparison, comparison_adj_mat) %>%
    mutate(
      gene_tf = map(comparison_adj_mat, map_gene_tf_network_analysis,
pipeline_input)
    )
}





#
map_gene_tf_network_analysis <- function(adj_mat, pipeline_input){

  previous <- getwd()
  setwd(paste0(getwd(), '/data/DREM/', pipeline_input$analysis_type))

  paths <- getwd() %>%
    list.files(pattern = 'path', full.names = T) %>%
    tibble(path_file = .) %>%
    dplyr::mutate(
      path_data = map(path_file, vroom, col_types = cols()),
      path_data = map(path_data, ~dplyr::select(.x, !contains('SPOT') &
!starts_with('H'))),
      path_data = map(path_data, ~set_colnames(.x, gsub("\\|.*","",
colnames(.x))))
    ) %>%
    use_series(path_data) %>%
    map(make_unique) %>%
    bind_rows()

  tf_list <- pipeline_input$tf_list

  gene_tf_network_analysis(paths, adj_mat, tf_list)

  setwd(previous)
}



make_unique <- function(tib){
  new_names <- tib %>%
    names() %>%
    make.unique('_')
  names(tib) <- new_names
  return(tib)

}




gene_tf_network_analysis <- function(drem_gene_tf, adj_mat, tf_list){
```

```
  ids = drem_gene_tf %>%
    use_series(target_id) %>%
    make.names(unique = T)

  drem_gene_tf <- as.data.frame(drem_gene_tf)
  #new_names <- drem_gene_tf[,1] %>% unique()
  rownames(drem_gene_tf) = ids
  drem_gene_tf <- drem_gene_tf[,-1]
  rr=drem_gene_tf[rowSums(drem_gene_tf)>2,]
  rr=rr[,colSums(rr)>2]


  g <- graph.incidence(rr)
  V(g)$color <- V(g)$type
  V(g)$color=gsub("FALSE","red",V(g)$color)
  V(g)$color=gsub("TRUE","blue",V(g)$color)
  tkp.id<-tkplot(g, edge.color="gray30", layout=layout_as_bipartite)

  tk_center(tkp.id)
  #tk_fit(tkp.id, width = 467, height = 567)
  #tk_rotate(tkp.id, degree = -90, rad = NULL)




  #centrality_drem[order(centrality_drem$types_drem, decreasing = TRUE),]

  #adj_m <- adj_mat[(rownames(adj_mat)%in% ids),]

  adjacancy_matrix_of_drem_TF <- adj_mat[(rownames(adj_mat) %in% ids),]

  #adjacency mtrix for drem TF data that matches with genes
  adjacancy_matrix_of_drem_TF =
adj_mat[,colnames(adj_mat)[colnames(adj_mat) %in%
colnames(drem_gene_tf)]]



  ath_tf_list=read.table(tf_list, header = TRUE)
  ath_tf_list=gsub("\\..*","",ath_tf_list$TF_ID)
  ath_tf_list=as.data.frame(unique(ath_tf_list))
  #colnames(ath_tf_list)="TFs"
  #adjacancy matrix of database TF data that matches with genes

adjacancy_matrix_of_database_TF=adj_mat[,colnames(adj_mat)[colnames(adj_m
at) %in% ath_tf_list]]


  data_tf_gene_corr <-
unique(cbind(adjacancy_matrix_of_database_TF,adjacancy_matrix_of_drem_TF)
)
```

```
  m <- data_tf_gene_corr
  source_node=c()
  target_node=c()
  correlation<-c()
  genes_names <- rownames(data_tf_gene_corr)
  tf_names<-colnames(data_tf_gene_corr)
  i<-genes_names[1:dim(m)[1]]
  j<-tf_names[1:dim(m)[2]]

  for(gene in i)
  {
    for(gen in j)
    {
      if(m[gene,gen]>0.5){
        source_node<-c(source_node,gene)
        target_node<-c(target_node,gen)
        correlation<-c(correlation,m[gene,gen])
      }
    }
  }


  NetworkData <- data.frame(source_node, target_node, correlation)

  net=NetworkData %>% filter(correlation > 0.5)
  net=net %>% filter(correlation != 1)

  net=unique(net)

  g <- graph.empty(directed = F)
  node.out <- unique(net$target_node) #stringsAsFactor = F in data frame
  node.in <- unique(net$source_node) #stringsAsFactor = F in data frame
  g <- graph.data.frame(net, directed = F)
  V(g)$type <- V(g)$name %in% net[,2] #the second column of edges is TRUE
type
  E(g)$weight <- as.numeric(net[,3])
  g

  rr=get.incidence(g,attr = "weight")

  V(g)$color <- V(g)$type
  V(g)$color=gsub("FALSE","red",V(g)$color)
  V(g)$shape=gsub("FALSE","square",V(g)$shape)
  V(g)$color=gsub("TRUE","blue",V(g)$color)
  tkplot(g, edge.color="gray30",edge.width=E(g)$weight,
layout=layout_as_bipartite)

  #finding histogram of node gree
  degree_nodes <- degree(g, mode="all")
  V(g)$size <- degree_nodes
  hist(degree_nodes, breaks=1:150, main="Histogram of node degree")
  degree.distribution <- degree_distribution(g, cumulative=T, mode="all")
```

```
  plot( x=0:max(degree_nodes), y=1-degree.distribution, pch=19, cex=1.2,
col="orange",

        xlab="Degree_of_nodes", ylab="Cumulative Frequency")




  #finding centralities
  types_drem <- V(g)$type
  deg_drem <- igraph::degree(g)
  bet_drem <- betweenness(g)
  clos_drem <- closeness(g)
  eig_drem <- eigen_centrality(g)$vector

  cent_df_drem <- data.frame(types_drem, deg_drem, bet_drem, clos_drem,
eig_drem)

  cent_df_drem[order(cent_df_drem$type_drem, decreasing = TRUE),]
  #finding clusters based on Edge betweenness
  ceb <- cluster_edge_betweenness(g)
  #finding hubs score of each gene
  hs <- hub_score(g, weights=NA)$vector

}









DREM_network_overlap <- function(p, d){
  # p: pipeline input
  # d: DEG file tibble




  # point to network directory
  nets <- p %>%
    extract2('DREM') %>%
    paste0('TFInput')
  # list potential networks to access

  net_full <- nets %>%
    list.files(full.names = T)
  # files without source target filtered away.
  net_full_tib <- net_full %>%
    tibble(f = .) %>%
    mutate(
      x = map_lgl(f, ~readLines(.x, n = 1) %>%
                    str_detect('TF\tGene\tInput')
```

```
      )
    ) %>%
    filter(x)

  nets <- net_full_tib %>%
    dplyr::select(f) %>%
    unlist(use.names = F)

  # invite user to select network
  message('Please pick an item (corresponding to a network) to overlap
differentially expressed genes with: ')
  # format menu to select from
  net_select <- nets %>%
    as_tibble(.) %>%
    rownames_to_column() %>%
    dplyr::rename('item' = rowname, 'network' = value)
  # print selection menu
  print(net_select)
  # input selection item
  chosen_item <- readline('Type item number: ')

  # extract network file name
  net <- net_select %>%
    spread(item, network) %>%
    dplyr::select(all_of(chosen_item))

  # extract full path to network chosen
  path_to_chosen_net <- net_full[str_detect(net_full, net %>%
unlist(use.names = F))]
  # check which tool
  edgeR_tool <- d %>%
    unlist(use.names = F) %>%
    first() %>%
    str_detect(pattern = 'edgeR')
  if (edgeR_tool){
    pval_filter <- 'adj.P.Val'
    summarise_by <- 'logFC'
    file_name_pattern <- 'clean_counts'

    # Select the directory which contains the count data from STAR.
    counts_data <- d %>%
      dplyr::slice(1) %>%
      first() %>%
      str_replace(pattern = 'edgeR.*', replacement =
'STAR/Feature_counts/')

    sk <- 1

  }
  if (!edgeR_tool){
    message('Unable to analyze Sleuth results.')
    #pval_filter <- 'qval'
    #summarise_by <- 'b'
```

```
  }
  # column id for removing extraneous chars.
  gene_col <- 'target_id'
  # source and target definitions as in network files.
  target_source <- 'TF'
  target <- 'Gene'
  clean_dm <-  p %>%
    extract2('design_matrix') %>%
    clean_design_matrix(., p)
  # load deg files
  summarised_differentially_expressed_gene_sets <- d %>%
    mutate(

      # Read in differentially expressed gene sets; filter based on p-val
cut-off
      de_gene_sets = map(deg_file, ~read_tsv(.x, col_types = cols()) %>%
                           drop_na() %>%
                           filter(!!sym(pval_filter) <= p %>%
extract2('significance_cutoff'))

      ),
      summarised_de_gene_sets = map(de_gene_sets,

~summarise_differential_expression(differential_expression_gene_set = .x,
gene_column = gene_col, mean_summerize = summarise_by)
      ),
      extract_title = map_chr(deg_file, extract_title_from_file_name)
    ) %>%
    dplyr::select(-de_gene_sets)
  # unzip if needed
  # if (str_detect(string = path_to_chosen_net, '.gz$')){
  #   unzip(zipfile = path_to_chosen_net)
  #   path_to_chosen_net <- path_to_chosen_net %>% str_remove('.gz')
  # }

  network <- read.delim(path_to_chosen_net) %>%
    dplyr::select(all_of(target_source), all_of(target))
  # Make network edges unique.
  edge_list <- network %>%
    mutate(
      !!target_source := !!sym(target_source) %>% map(extract_edges)
    ) %>%
    unnest(cols = c(target_source)) %>%
    mutate(
      !!target := !!sym(target) %>% map_chr(extract_edges)
    )

  # Extract unique source nodes.
  unique_sources <- edge_list %>%
    unique_and_relabel('label_name' = target_source)

  # Extract unique target nodes.
  unique_targets <- edge_list %>%
    unique_and_relabel(label_name = target)
```

```
  # Combine all unique sources and all unique targets and label each a
unique identifier.
  all_nodes <- full_join(unique_sources, unique_targets, by = 'label')
%>%
    rowid_to_column('id')

  # Associate unique gene ids with edges.
  edge_list_ids <- edge_list %>%
    left_join(all_nodes, by = setNames(nm = target_source, 'label')) %>%
    dplyr::rename(from = id) %>%
    left_join(all_nodes, by = setNames(nm = target, 'label')) %>%
    dplyr::rename(to = id)


  # Number of columns the extracted title can be divided into.
  column_number <-
extract_condition_column_number(extracted_title_dataset =
summarised_differentially_expressed_gene_sets)

  # Group comparison set elements.
  comparison_elements <-
associate_comparison_elements(extracted_title_dataset =
summarised_differentially_expressed_gene_sets, number_of_columns =
column_number)

  # Associate expression data (counts) replicate sets with conditions.
  associate_expression_replicates <-
associate_replicate_sets_to_conditions(expression_data_location =
counts_data, clean_dm, sk, p)

  # Associate replicate sets to differential gene expression comparison
sets.
  associate_expression_values_to_comparison_sets <-
associate_expression_to_comparison_elements(replicate_set_association =
associate_expression_replicates, comparisons = comparison_elements)

  # Uniquely name the column in the large tibble to contain overlapped
nodes.
  overlap_sources_and_targets_column <- paste(target_source, target, sep
= '_and_')


  # Associate read count data (expression data) with comparison sets
(comparison elements).
  comparison_set_expression <-
associate_expression_values_to_comparison_sets %>%
    mutate(

      # Paste groups together to join with extract_title column in
node_overlap variable.
      extract_title = map2_chr(group1, group2,
paste_groups_to_join_with_title_extract),
```

```
      # Take the mean expression value for replicates, and the mean
expression value across each comparison.
      gene_expression = map2(group1_replicate_data,
group2_replicate_data, mean_replicate_est_counts)
    )


  # Overlap differentially expressed genes with network edges.
  node_overlap <- summarised_differentially_expressed_gene_sets %>%

    # remove '.tsv'

    mutate(
      extract_title = str_remove(extract_title, pattern = '\\..*') %>%
        str_remove('_vs')
    ) %>%

    dplyr::right_join(comparison_set_expression, by = 'extract_title')
%>%

    dplyr::select(-contains('group')) %>%

    mutate(


      # Extract read count data to associate along side differential
expression measurement.
      summarised_de_gene_sets = map2(summarised_de_gene_sets,
gene_expression, ~inner_join(.x, .y, by = gene_col)),


      # Collect target sources found in each differentially expressed
gene set.
      !!overlap_sources_and_targets_column :=
map(summarised_de_gene_sets,
~overlap_sources(differential_gene_expression_set = .x, edges =
edge_list_ids, node_type = target_source, summarise_col = summarise_by)),


      # Collect target measurements similarly.
      !!overlap_sources_and_targets_column :=
map2(summarised_de_gene_sets, !!sym(overlap_sources_and_targets_column),
~overlap_targets(differential_gene_expression_set = .x, target_sources =
.y, node_type = target, summarise_col = summarise_by)),


      # Adjacency matrix for each network.
      adjacency_matrix = map(!!sym(overlap_sources_and_targets_column),
get_adjacency_matrix),


      # Collect edge information.
      edges = map(!!sym(overlap_sources_and_targets_column),
~dplyr::select(.x, matches('from|to'))),
```

```
      # ID the nodes within each set.
      relabel_nodes = map(!!sym(overlap_sources_and_targets_column),
~relabel_local_nodes(local_edges = .x, target_sources = target_source,
targets = target)),


      # Map beta values to unique genes.
      map_betas_to_nodes = map(!!sym(overlap_sources_and_targets_column),
~beta_and_read_mapping_to_nodes(target_source_set = .x, node_type_1 =
target_source, node_type_2 = target, summarise_col = summarise_by)),


      # Label each identifier as either target or source.
      label_type = map(map_betas_to_nodes,
~label_and_rescale_mapped_values(mapped_values_set = .x, edges =
edge_list, first_label = target_source, second_label = target))


      # Extract in-degree and out-degree for each node in a network.
      #node_degree = map2(label_type, adjacency_matrix,
~map_degrees_to_nodes(node_information = .x, adj_matrix = .y,
node_universe = all_nodes))

    )

  tmp <- getwd() %>% paste0('/data/', p$analysis_type, '_DEG_networks')
  dir.create(tmp, recursive = T)

  expression_networks <- node_overlap %>%
    mutate(
      #
      graph_data = pmap(list(map_betas_to_nodes, edges, label_type,
extract_title), graph_differential_gene_expression_network),

      #
      write_graph_data = map2(extract_title, graph_data,
~paste_and_save(output_directory = tmp, file_name_to_paste = .x, graph =
.y))
    )
}






graph_differential_gene_expression_network <- function(nodes, edges,
label_types, titles_extracted) {

  as_tbl_graph(nodes, edges %>% unlist()) %>%
    activate(nodes) %>%
    left_join(label_types, by = 'name') %>%
```

```
    dplyr::rename('Identifier' = name) %>%
    dplyr::rename('Class' = label) %>%
    ggraph(layout = 'kk') +
    geom_edge_link(arrow = arrow(length = unit(3, 'mm'))) +
    geom_node_point(aes(alpha = scaled_beta, colour = Identifier, size =
reads, shape = Beta_coefficient)) +
    geom_node_text(aes(label = Class), color = 'black', size = 2, vjust =
2, show.legend = FALSE) +
    theme_graph() +
    ggtitle(titles_extracted) +
    labs(alpha = 'Scaled LFC value', size = 'Mean of counts', shape =
'LFC') +
    guides(color = FALSE)
}



paste_and_save <- function(output_directory, file_name_to_paste, graph){
  output_directory <- output_directory %>%
    paste0('/', file_name_to_paste, '.png')

  graph %>%
    ggsave(filename = output_directory, device = 'png', dpi = 320, width
= 10.00, height = 10.00, units = 'in')

}



label_and_rescale_mapped_values <- function(mapped_values_set, edges,
first_label, second_label){

  # Extract value range for scaling.
  rescale_set <- mapped_values_set %>%
    dplyr::select(beta) %>%
    range()


  mapped_values_set <- mapped_values_set %>%
    mutate(

      # Rescale values for graphing with alpha.
      scaled_beta = map_dbl(beta, ~rescale(x = .x, from = rescale_set, to
= c(0,1))),

      # Label beta coefficient.
      Beta_coefficient = map_chr(beta, ~if_else(condition = (.x < 0),
                                                true = '-',
                                                false = '+'))

    )

  mapped_values_set %>%
    left_join(x = ., y = edges, by = c('name' = first_label)) %>%
```

```
    dplyr::rename(!!first_label := second_label) %>%


    mutate_at(first_label, ~if_else(condition = is.na(.),
                                    true = replace(x = ., values =
second_label),
                                    false = replace(x = ., values =
first_label)
    )
    ) %>%
    dplyr::rename(label := !!first_label) %>%
    distinct()
}
```

```
beta_and_read_mapping_to_nodes <- function(target_source_set,
node_type_1, node_type_2, summarise_col){
  node_type_1_set <- c(node_type_1, paste0(node_type_1, '_',
summarise_col), paste0(node_type_1, '_mean_counts'))
  node_type_2_set <- c(node_type_2, paste0(node_type_2, '_',
summarise_col), paste0(node_type_2, '_mean_counts'))

  target_source_set %>%
    nest(data = c(node_type_1_set, node_type_2_set)) %>%
    dplyr::rename(tmp := data) %>%
    dplyr::select(tmp) %>%
    mutate(
      tmp = map(tmp, gather)
    ) %>%
    unnest(cols = c(tmp)) %>%
    dplyr::select(value) %>%
    mutate(
      ind = rep(c(1,2,3), length.out = n())
    ) %>%
    group_by(ind) %>%
    mutate(
      id = row_number()
    ) %>%
    spread(ind, value) %>%
    dplyr::select(-id) %>%
    dplyr::rename(name = '1', beta = '2', reads = '3') %>%
    mutate_at(vars(beta, reads), as.numeric) %>%
    distinct()
}



relabel_local_nodes <- function(local_edges, target_sources, targets){
```

```
  local_edges %>%
    dplyr::select(target_sources, targets) %>%
    unlist(use.names = FALSE) %>%
    tibble(name = .) %>%
    distinct() %>%
    rowid_to_column('id')
}


get_adjacency_matrix <- function(overlap_sources_and_targets_column){
  overlap_sources_and_targets_column %>%
    dplyr::select(to, from) %>%
    as.data.frame() %>%
    graph.data.frame() %>%
    get.adjacency() %>%
    as.matrix()
}



overlap_targets <- function(differential_gene_expression_set,
target_sources, node_type, summarise_col){
  differential_gene_expression_set %>%
    dplyr::rename(!!node_type := target_id) %>%
    right_join(target_sources, by = node_type) %>%
    drop_na() %>%
    dplyr::rename(!!paste0(node_type, '_', summarise_col) :=
paste0(summarise_col, '_mean')) %>%
    dplyr::rename(!!paste0(node_type, '_mean_counts') := mean_counts)
}




overlap_sources <- function(differential_gene_expression_set, edges,
node_type, summarise_col){
  differential_gene_expression_set %>%
    dplyr::rename(!!node_type := target_id) %>%
    left_join(edges, by = node_type) %>%
    drop_na() %>%
    dplyr::rename(!!paste0(node_type, '_', summarise_col) :=
paste0(summarise_col, '_mean')) %>%
    dplyr::rename(!!paste0(node_type, '_mean_counts') := mean_counts)
}




average_counts_across_comparison_sets <- function(est_count_set){
  est_count_set %>%
    dplyr::rename(target_id = 1, counts = 2, counts1 = 4) %>%
    dplyr::select(ends_with('id'), contains('counts')) %>%
    mutate(
      mean_counts = map2_dbl(counts, counts1, ~mean(x = c(.x, .y)))
```

```
    ) %>%
    dplyr::select(target_id, mean_counts)
}


average_est_counts_for_replicates <- function(group){
  group %>%
    unnest(cols = c(count_data)) %>%
    group_by(target_id) %>%
    summarise(
      counts = mean(counts, na.rm = TRUE)
    )
}

mean_replicate_est_counts <- function(column_one, column_two){

  column_one <- column_one %>% average_est_counts_for_replicates()
  column_two <- column_two %>% average_est_counts_for_replicates()
  bind_cols(column_one, column_two) %>%
    average_counts_across_comparison_sets(est_count_set = .)
}



paste_groups_to_join_with_title_extract <- function(column_one,
column_two){
  column_one %>%
    paste(column_two, sep = ' ') %>%
    str_replace_all(pattern = ' ', replacement = '_')
}


associate_expression_to_comparison_elements <-
function(replicate_set_association, comparisons){
  comparisons %>%
    inner_join(replicate_set_association, by = c('group1' = 'condition'))
%>%
    dplyr::rename(group1_replicate_data = replicate_data) %>%

    left_join(replicate_set_association, by = c('group2' = 'condition'))
%>%
    dplyr::rename(group2_replicate_data = replicate_data)
}




associate_comparison_elements <- function(extracted_title_dataset,
number_of_columns){

  start <- (number_of_columns - number_of_columns) + 1
  mid <- number_of_columns/2
  mid_right <- round(mid) + 1
```

```
  extracted_title_dataset %>%
    dplyr::select(extract_title) %>%
    tidyr::extract(col = extract_title,
                   into = rep('id', times = number_of_columns) %>%
paste(1:number_of_columns, sep = ''),
                   regex = rep('(.*)', times = number_of_columns) %>%
paste(collapse = '_')) %>%

    unite(col = group1, rep('id', times = (mid)) %>% paste0(start:mid),
sep = ' ') %>%
    unite(col = group2, rep('id', times = (mid)) %>%
paste0(mid_right:number_of_columns), sep = ' ') %>%
    mutate(
      group2 = str_remove(group2, '.tsv')
    )
}


clean_design_matrix <- function(dm, p){
  #p: pipeline input
  covars <- p$sample_covariates
  dm %>%
    read_csv(col_types = cols()) %>%
    tidyr::extract(condition,
                   into = str_split(pattern = ', ', covars) %>% unlist(),
                   regex = '(.*) (.*) ([[:digit:]].*)')
}


associate_replicate_sets_to_conditions <-
function(expression_data_location, clean_dm, sk, p){
  tmp <- str_split(pattern = ', ', p$sample_covariates) %>% unlist()
  expression_data <- expression_data_location %>%
    tibble(files = list.files(path = ., full.names = TRUE, recursive =
TRUE)) %>%

    mutate(

      files = map_chr(files, ~str_replace(.x, pattern = '//', replacement
= '/')),
      count_data = map(files,
                       read_tsv_filter_extraneous,
                       sk
      ),
      sample = map_chr(files, ~basename(.x) %>% str_remove('\\_.*'))
    ) %>%


    # Join by sample identifiers.
    left_join(y = clean_dm, by = 'sample') %>%
    dplyr::select(files, count_data, sample, all_of(tmp)) %>%
    tidyr::unite(col = condition, tmp, sep = ' ') %>%
    dplyr::select(count_data, condition) %>%
    group_by(condition) %>%
```

```
    nest() %>%
    dplyr::rename(replicate_data = data)
}



read_tsv_filter_extraneous <- function(current, s){
  # current: current counts file
  # s: skip lines
  current %>%
    read_tsv(file = ., col_names = T, skip = s, col_types = cols()) %>%
    dplyr::rename(target_id = 1, est_counts = 2) %>%
    mutate(
      target_id = str_remove(string = target_id, '\\..*')
    ) %>%
    group_by(target_id) %>%
    summarise(counts = mean(est_counts)) %>%
    ungroup()
}



associate_expression_to_comparison_elements <-
function(replicate_set_association, comparisons){
  comparisons %>%
    inner_join(replicate_set_association, by = c('group1' = 'condition'))
%>%
    dplyr::rename(group1_replicate_data = replicate_data) %>%

    left_join(replicate_set_association, by = c('group2' = 'condition'))
%>%
    dplyr::rename(group2_replicate_data = replicate_data)
}



extract_condition_column_number <- function(extracted_title_dataset){
  extracted_title_dataset %>%
    dplyr::select(extract_title) %>%
    mutate(
      column_number = map_dbl(extract_title, ~str_count(.x, '_') %>%
add(1) %>% as.numeric())
    ) %>%
    dplyr::select(column_number) %>%
    unlist(use.names = FALSE) %>%
    unique()
}



unique_and_relabel <- function(edges, label_name){
  edges %>%
    distinct(!!sym(label_name)) %>%
    dplyr::rename(label = !!label_name)
```

```
}

extract_edges <- function(field){
  field %>%
    str_split(pattern = '\\|') %>%
    unlist() %>%
    unique()
}

# remove extraneous chars in gene column. summarise chosen column by mean
of values in column.
summarise_differential_expression <-
function(differential_expression_gene_set, gene_column, mean_summerize){
  differential_expression_gene_set %>%
    mutate(
      !!gene_column := map_chr(!!sym(gene_column), ~str_remove(.x,
pattern = '\\..*'))
    ) %>%
    group_by(!!sym(gene_column)) %>%
    summarize(!!sym(paste0(mean_summerize, '_mean')) :=
mean(!!sym(mean_summerize), na.rm = T))
}

extract_title_from_file_name <- function(file_name){
  file_name %>%
    basename() %>%
    str_remove('_results[[:punct:]].*')
}




# Function name: wgcna_input_data
# Purpose: Takes the input data and convert it into
#          differentially expreesed expression data for wgcna analysis.
# Input: path to differentially expressed genes and pipeline input.
# Output: filtered count data.
wgcna_input_data <- function(differentialy_expressed_genes,
pipeline_input){

  # Load design matrix into environment.
  design_matrix <- pipeline_input$design_matrix %>%
    read_csv(col_types = cols())

  # Detect whether analyzing differentially expressed genes from
  # edgeR or Sleuth.
  edgeR_tool <- differentialy_expressed_genes %>%
    str_detect(pattern = 'edgeR')

  # Select the column which contains the p-values or q-values
  # depending on which differential expression tool was selected.
  if(edgeR_tool){
```

```
    # The edgeR analysis output has 'adj.P.Val' as the p-value
    # column name.
    filter_value <- 'adj.P.Val'

    # Counts data from feature counts contains the 'clean_counts'
    # string in the nane.
    file_name_pattern <- 'clean_counts'

    # Select the directory which contains the count data from STAR.
    counts_data <- differentialy_expressed_genes %>%
      str_replace('edgeR.*', 'STAR/Feature_counts/')

  }
  else if(!edgeR_tool){

    # Sleuth has q-values in the 'qval' column.
    filter_value <- 'qval'

    # Counts data are in TSV files from Kallisto.
    file_name_pattern <- '^abundance.tsv$'

    counts_data <- differentialy_expressed_genes %>%
      str_replace('Sleuth.*', 'Kallisto/Kallisto_quantifications/')
  }

  # Load read count data and filter lowly expressed genes.
  keep_counts <- counts_data %>%

    # Read count data into environment; use explicit patterns
    # which allow distinctions between counts datasets.
    counts_keep(file_name_pattern, recurse_subdirectories = !edgeR_tool)
%>%

    # Map count data to sample names from design matrix.
    #associate_counts_with_sample_names(design_matrix) %>%

    # Filter lowly expressed genes from count data.
    filter_counts_data_wgcna(counts = ., design_matrix = design_matrix)
%>%

    mutate(
      target_id = map(target_id,
                      ~gsub(pattern = '\\..*', replacement = '', x = .x)
      ),

      target_id = target_id %>%
        as.character()
    )




  # Load differential gene expression data.
```

```
  differentially_expressed_genes_keep(differentialy_expressed_genes,
filter_value, pipeline_input) %>%
    mutate(
      original_filter = deg_data2,
      deg_data2 = map(deg_data2,
                      ~suppressMessages(semi_join(keep_counts, .x))
      ),
      preprocess_wgcna_input = map(deg_data2,
                                   wgcna_data_processing
      )
    )


}




# Function name: counts_keep
# Purpose: Load the count data into the environment using decisions
#          from which count data type is being sourced.
# Input: Counts data, file name regex, Boolean value.
# Output: Counts data from files.
counts_keep <- function(feature_counts, file_name_pattern,
recurse_subdirectories){

  # Skip lines for feature counts data, but not for Kallisto data.
  if(recurse_subdirectories){
    nrow_skip <- 0
  } else{
    nrow_skip <-  1
  }

  # Load count data into environment based on the file name pattern.
  list.files(feature_counts, pattern = file_name_pattern, full.names =
TRUE, recursive = recurse_subdirectories) %>%
    tibble(files = .) %>%

    # Allow column names to be passed from data source and skip a number
of rows.
    mutate(
      sample_count_data = map(files, read_tsv, skip = nrow_skip,
col_names = TRUE, col_types = cols())
    )
}




# Function name: filter_counts_data_wgcna
# Purpose: Filter the lowly expressed genes from the data
#          obtained from feature counts.
# Input: Counts data and the experimental design matrix.
# Output: Counts mapped to sample in a dataframe.
```

```
filter_counts_data_wgcna <- function(counts, design_matrix){

  # Check for Kallisto data.
  is_kallisto <- counts %>%
    dplyr::slice(1) %>%
    dplyr::select(files) %>%
    str_detect('Kallisto_quantifications')

  # Cut extra statistics, leaving only the count data
  # for genes.
  if(is_kallisto){
    gene_names <- counts %>%
      dplyr::slice(1) %>%
      dplyr::select(sample_count_data) %>%
      unnest(sample_count_data) %>%
      dplyr::select(gene)

    counts <- counts %>%
      mutate(
        sample_count_data = purrr::map(sample_count_data,
reset_splice_variants, gene_names = gene_names)
      )
  }

  samplename <- design_matrix

  data <- data.frame(counts$sample_count_data)

  # Storing data as data frame.
  total_samples <- nrow(counts)*2
  ans <- seq(2,total_samples,2)
  data = data[c(1,ans)]

  #(optional) naming the samples
  #d=colnames(sleuth_table)[1]
  d = "target_id"
  x <- samplename$sample
  a <- c(d,x)
  colnames(data) <- a
  rowname <- data[c(1)]

  #filterng the data
  keep <- rowSums(cpm(data[,2:length(data)]) > 0.5) >= 2

  data[keep,]
}




# Function name: reset_splice_variants
# Purpose: Remove splice variant notation.
# Input: Count data and unique gene names.
# Output: Gene names without spliced notation.
```

```
reset_splice_variants <- function(sample_counts, gene_names){
  sample_counts %>%
    mutate(
      target_id = gene_names %>%
        unlist(use.names = F)
    ) %>%
    dplyr::select(target_id, est_counts)
}




# Function name: wgcna_data_processing
# Purpose: Preprocess the input data for wgcna.
# Input: Count data specific to a comparison set from
#        differential gene expression testing.
# Output: Transposed count data.
wgcna_data_processing <- function(comparison_set_counts){

  Expression_data0 <- comparison_set_counts[, -c(1)]
  Expression_data0 <- as.data.frame(t(Expression_data0))
  names(Expression_data0) = comparison_set_counts$target_id
  goodsamples = goodSamplesGenes(Expression_data0, verbose = 0)
  if (!goodsamples$allOK)
  {
    if (sum(!goodsamples$goodGenes)>0)
      printFlush(paste("Removing genes:",
paste(names(Expression_data0)[!goodsamples$goodGenes], collapse = ",
")));
    if (sum(!goodsamples$goodSamples)>0)
      printFlush(paste("Removing samples:",
paste(rownames(Expression_data0)[!goodsamples$goodSamples], collapse = ",
")));
    Expression_data0 = Expression_data0[goodsamples$goodSamples,
goodsamples$goodGenes]
  }
  Expression_data0 <- as.data.frame(Expression_data0)
  return(Expression_data0)
}




# Function name: differentially_expressed_genes_keep
# Purpose: Find the count data of differentially expressed
#          genes from TSV files of EdgeR/ Sleuth results
#          regardless of which tool was used.
# Input: DGE data directory, p-value, and pipeline input structure.
# Output: Genes filtered with the cut-off (p-value).
differentially_expressed_genes_keep <- function(deg_directory,
filter_value, pipeline_input){
  # Count data files are TSV files regardless of which tool was used.
```

```
  tibble(files = list.files(deg_directory, pattern = '.tsv', full.names =
TRUE)) %>%
    mutate(
      comparison = map_chr(files,
                           extract_comparison
      ),
      deg_data2 = map(files,
                      read_and_filter,
                      filter_value,
                      pipeline_input
      )
    )
}




# Function name: extract_comparison
# Purpose: Find the comparision file name and remove .tsv string pattern
# Input: Filename for a differential gene expression test result.
# Output: Input without .tsv extension.
extract_comparison <- function(comparison_filename){
  comparison_filename %>%
    basename() %>%
    str_remove('.tsv')
}



# Function name: read_and_filter
# Purpose: Reads the data from Sleuth/ EdgeR and
#          filters out the differentially expressed genes.
# Input: Set of differentially expressed genes, cut-off value,
#        and pipeline input structure.
# Output: The input set filtered on the cut-off value.
read_and_filter <- function(differentialy_expressed_genes_sets,
filter_value, pipeline_input){
  differentialy_expressed_genes_sets %>%
    read_tsv(col_types = cols()) %>%
    drop_na() %>%
    filter(!!sym(filter_value) <= pipeline_input$significance_cutoff) %>%
    dplyr::select(target_id)
}





# Function name: wgcna_plot_sample_tree
# Purpose: Plot to find any outlier sample in the data
#          for all the comparisions.
# Input: Expression_data0.
```

```
# Output: Plotted sample tree.
wgcna_plot_sample_tree <- function(Expression_data0){
  Expression_data0 %>%
    mutate(
      sample_tree = map(preprocess_wgcna_input,
                        hclust_distance_matrix,
                        'average'
      ),

      # Plot the sample tree:
      sample_clustering = purrr::pmap(
        list(sample_tree,
             preprocess_wgcna_input,
             files,
             comparison),
        plot_sample_tree
      )
    )
}




# Function name: hclust_distance_matrix
# Purpose: Find the distance matrix to perform clustering.
# Input: Preprocessed WGCNA data and the 'average' method.
# Output: Analyzed hierarchical cluster.
hclust_distance_matrix <- function(Expression_data0, method){
  Expression_data0 %>%
    dist() %>%
    hclust(method)
}




# Function name: plot_sample_tree
# Purpose: Plot to find any outlier sample in the data .
# Input: hclust result, preprocessed WGCNA data, file name from
#        differential gene expression test, and the names of
#        comparisons made.
# Output: List: datExp, sft, power, and k values.
plot_sample_tree <- function(sample_tree, Expression_data0, file_name,
comparison){
  sizeGrWindow(12,9)
  par(cex = 0.6);
  par(mar = c(0,4,2,0))

  p.plot <- sample_tree %>% plot(main = "Sample clustering to detect
outliers", sub="", xlab="", cex.lab = 1.5, cex.axis = 1.5, cex.main = 2)

  choose_line <- readline(prompt = "Enter the number at which the limit
that shoud be cut or remove the outlier Hint:the sample that seems to be
the outlier: ")
```

```
  choose_line <- as.integer(choose_line)

  if (is.na(choose_line)){
    print("Enter a valid number.")
  }


  sample_clust <- sample_tree %>%
    cutreeStatic(cutHeight = choose_line, minSize = 10)

  keepSamples <- (sample_clust==1)

  datExpr <- Expression_data0[keepSamples, ]
  powers <-  c(c(1:10), seq(from = 12, to=100, by=2))
  invisible(capture.output(sft <-  pickSoftThreshold(datExpr, powerVector
= powers, verbose = 0, networkType = "signed")))
  power <-
sft$fitIndices$Power[which(sft$fitIndices$SFT.R.sq==max(sft$fitIndices$SF
T.R.sq))]
  k <- softConnectivity(datE = datExpr, power = power, verbose = 0)

  return(list(datExpr = datExpr, sft = sft, power = power, k = k))
}



# Function name: wgcna_plot_power_results
# Purpose: Histogram plot to analyse and choose
#          correct power value for all the comparisions
# Input: Column which contains the sft values.
# Output: Plot in Rstudio Plots pane.
wgcna_plot_power_results <- function(contains_sft){
  contains_sft %>%
    mutate(
      power_results = map2(sample_clustering,
                           comparison,
                           plot_power_results)
    )
}




# Function name: plot_power_results
# Purpose: Histogram plot to analyse and choose correct power value.
# Input: datExp, sft, power, and k values; comparison names.
# Output: Analysis for scale-free topology and mean connectivity.
plot_power_results <- function(sample_clustering, comparison){
  comparison <- comparison %>%
    str_replace_all('_', ' ') %>%
    paste('Analyzing', .)

  print(comparison)
```

```
  sft <- sample_clustering %>%
    use_series(sft)

  sizeGrWindow(9, 5)
  par(mfrow = c(1,2));
  cex1 = 0.9;
  powers = c(c(1:10), seq(from = 12, to=100, by=2))
  plot(sft$fitIndices[,1], -sign(sft$fitIndices[,3])*sft$fitIndices[,2],
       xlab="Soft Threshold (power)",ylab="Scale Free Topology Model
Fit,signed R^2",type="n",
       main = paste("Scale independence"));
  text(sft$fitIndices[,1], -sign(sft$fitIndices[,3])*sft$fitIndices[,2],
       labels=powers,cex=cex1,col="red");

  abline(h=0.90,col="red")

  p1.plot<-plot(sft$fitIndices[,1], sft$fitIndices[,5],
                xlab="Soft Threshold (power)",ylab="Mean Connectivity",
type="n",
                main = paste("Mean connectivity"))
  text(sft$fitIndices[,1], sft$fitIndices[,5], labels=powers,
cex=cex1,col="red")

  readline(prompt="Press [enter] to see next figure. ")
}




# Function name: wgcna_plot_power_histogram
# Purpose: Plot to choose the correct power
#          from scale free topology for all the comparisions.
# Input: Column with k values.
# Output: Plot in Rstudio Plots pane.
wgcna_plot_power_histogram <- function(contains_k){
  contains_k %>%
    mutate(
      power_histogram_results = map2(sample_clustering,
                                     comparison,
                                     plot_power_histogram)
    )
}




# Function name: plot_power_histogram
# Purpose: Plot to choose the correct power
#          from sclae free topology.
# Input: datExp, sft, power, and k values; comparison names.
# Output: Scale free topology plot.
plot_power_histogram <- function(sample_clustering, comparison){
```

```
  comparison <- comparison %>%
    str_replace_all('_', ' ') %>%
    paste('Analyzing', .)

  print(comparison)

  k <- sample_clustering %>%
    use_series(k)


  sizeGrWindow(10,5)
  par(mfrow=c(1,2))
  p3.plot<-hist(k)
  p4.plot<-scaleFreePlot(k, main="Check scale free topology\n")

  readline(prompt = "Press [enter] to see next figure. ")

  return(list(p3.plot, p4.plot))

}



# Function name: wgcna_clustering
# Purpose: Perform clustering and saves result in their respective
directory.
# Input: datExp, sft, power, and k values.
# Output: Plot in Rstudio Plots pane.
wgcna_clustering <- function(sample_clustering){
  sample_clustering %>%
    mutate(
      clustering_res = map2(sample_clustering,
                            files,
                            clustering)
    )

}





# Function name: wgcna_clustering
# Purpose: Perform clustering and saves result in their respective
directory.
# Input: datExp, sft, power, and k values; file path to write dendrograms
to.
# Output: datExp, dynamic_Modules, adjacency, and dynamic_Colors values.
clustering <- function(sample_clustering, file_path){

  cluster_dend_dir <- file_path %>%
    dirname() %>%
    paste0('/cluster_dendrograms/') %>%
    str_replace(pattern = '//', replacement = '/')
```

```
  dir.create(cluster_dend_dir, showWarnings = FALSE, recursive = TRUE)

  file_path <- file_path %>%
    basename() %>%
    paste0(cluster_dend_dir, .) %>%
    str_replace('.tsv$', '.pdf')

  datExpr <- sample_clustering %>%
    use_series(datExp)

  sft <- sample_clustering %>%
    use_series(sft)

  softPower <- sample_clustering %>%
    use_series(softPower)

  k <- sample_clustering %>%
    use_series(k)


  adjacency = adjacency(datExpr, power = 12)

  # topological overlap matrix
  TOM = TOMsimilarity(adjacency, verbose = 0)
  dissTOM = 1-TOM

  # hierarchical clustering function
  geneTree = hclust(as.dist(dissTOM), method = "average")
  minModuleSize = 30
  dynamic_Modules = cutreeDynamic(dendro = geneTree, distM = dissTOM,
                                deepSplit = 2, pamRespectsDendro =
FALSE,
                                minClusterSize = minModuleSize, verbose
= 0)
  #convert colors

  dynamic_Colors = labels2colors(dynamic_Modules)


  Module_eigenList = moduleEigengenes(datExpr, colors = dynamic_Colors)
  Module_eigenvals = Module_eigenList$eigengenes
  Module_eigenDiss = 1-cor(Module_eigenvals)


  module_eigenTree = flashClust(as.dist(Module_eigenDiss), method =
"average");
  Module_eigenDissThres = 0.25

  merge = mergeCloseModules(datExpr, dynamic_Colors, cutHeight =
Module_eigenDissThres, verbose = 0)


  mergedColors = merge$colors;
```

```
  sizeGrWindow(12, 9)
  pdf(file = file_path, wi = 9, he = 6)
  p.plot <- plotDendroAndColors(geneTree, cbind(dynamic_Colors,
mergedColors),
                                c("Dynamic Tree Cut", "Merged dynamic"),
                                dendroLabels = FALSE, hang = 0.03,
                                addGuide = TRUE, guideHang = 0.05)
  dev.off()


  mergedMEs = merge$newMEs

  return(list(datExpr = datExpr,
              dynamic_Modules = dynamic_Modules,
              adjacency = adjacency,
              dynamic_Colors = dynamic_Colors))

}



# Function name: implement_GO_enrichment
# Purpose: Checks the DEG data on which GO analysis to be performed
#          and performs GO analysis.
# Input: String specifying which tool was used; alignment results
#        structure.
# Output: GO enrichment results.
implement_GO_enrichment <- function(deg_tool, alignment_results){

  alignment_decision <- alignment_results %>%
    dplyr::first()

  pipeline_input <- alignment_results %>%
    dplyr::last()

  if(deg_tool %>% str_detect('Sleuth')){
    deg_input <- alignment_decision %>%
      filter(key == 'kallisto') %>%
      dplyr::select(directories) %>%
      unlist(use.names = FALSE) %>%
      first() %>%
      str_replace('Kallisto', paste0('Sleuth/',
pipeline_input$analysis_type, '/gene_level/gene_level_results'))

    pattern <- '.tsv$'

    filter_value <- 'qval'

    GO_enrichment <- enrichment(deg_directory = deg_input, pattern =
pattern)
  }

  if(deg_tool %>% str_detect('edgeR')){
```

```
    deg_input <- pipeline_input$star_genomeDir %>%
      str_replace('STAR.*', paste0('edgeR/',
pipeline_input$analysis_type, '/')) %>%
      str_replace(pattern = '//', replacement = '/')

    pattern <- '.tsv$'

    filter_value <- 'adj.P.Val'

    GO_enrichment <- enrichment(deg_directory = deg_input, pattern =
pattern, pipeline_input, filter_value)

  }

}




# Function name: enrichment
# Purpose: Test for significantly enriched genes in the deg gene sets.
# Input: Directory to deg files, file name pattern, pipeline input, and
#        filter value.
# Output: Go enrichment.
enrichment <- function(deg_directory, pattern, pipeline_input,
filter_value){


  deg_file_tib <- list.files(path = deg_directory, pattern = pattern,
full.names = TRUE, recursive = TRUE) %>%
    tibble(deg_file = .) %>%
    mutate(
      deg_data = map(deg_file,
                     read_tsv,
                     col_types = cols()
      )
    )


  mart_arabdopsis <- biomaRt::useMart(biomart = "plants_mart",
                                      dataset = "athaliana_eg_gene",
                                      host = 'plants.ensembl.org',
                                      verbose = FALSE)


  Gene_go <- suppressMessages(biomaRt::getBM(attributes = c(
"ensembl_gene_id", "go_id"), mart = mart_arabdopsis, verbose = F))

  geneID2GO <- by(Gene_go$go_id,
                  Gene_go$ensembl_gene_id,
                  function(x) as.character(x))

  filename_for_go <- extract_filename_go(deg_file_tib)
  go_input <- deg_file_tib$deg_data
```

```
  tmp <- list()
  gene_name <- list()
  result_GO_gene <- list()
  for (i in 1:length(go_input))
  {
    tmp[[i]] <- go_input[[i]] %>%
      filter(!!sym(filter_value) <= pipeline_input$significance_cutoff)

    gene_name[[i]] <- tmp[[i]] %>%
      dplyr::select("target_id")

    if (filter_value == 'pval') {
      tmp[[i]] <- tmp[[i]] %>%
        dplyr::select( 'pval' )
    } else {
      tmp[[i]] <- tmp[[i]] %>%
        dplyr::select( 'adj.P.Val' )
    }


    geneList <- as.numeric(unlist(tmp[[i]]))
    names(geneList) <- as.character(unlist(gene_name[[i]]))

    # Create topGOData object
    GOdata <- suppressMessages(
      new("topGOdata",
          ontology = "BP",
          allGenes = geneList,
          geneSelectionFun = function(x)(x == 1),
          annot = annFUN.gene2GO, gene2GO = geneID2GO)
    )

    # Kolmogorov-Smirnov testing
    result_KS <- suppressMessages(
      runTest(GOdata, algorithm = "weight01", statistic = "ks")
    )

    GO_result_tab <- GenTable(GOdata, raw.p.value = result_KS, topNodes =
length(result_KS@score), numChar = 120)

    par(cex = 1)

    showSigOfNodes(GOdata, score(result_KS), firstSigNodes = 10, useInfo
= "def")

    print(head(GO_result_tab))

    printGraph(GOdata, result_KS, firstSigNodes = 10, fn.prefix = "tGO",
useInfo = "all", pdfSW = TRUE)

    my_GO_term <- c(GO_result_tab$GO.ID[1])
    my_genes <- genesInTerm(GOdata, my_GO_term)
```

```
    for (j in 1:length(my_GO_term))
    {
      my_GO_term2 <- my_GO_term[j]
      my_genes_GO_term <- my_genes[my_GO_term2][[1]]
      my_genes_GO_term <- paste(my_genes_GO_term, collapse=',')
      print(paste("Term",my_GO_term,"genes:", my_genes_GO_term))

    }
    result_GO_gene[i] <- my_genes_GO_term
  }
  return(list(result_GO_gene = result_GO_gene, filename_for_go =
filename_for_go))
}



# Function name: extract_filename_go
# Purpose: Extracts the comparision set for which
#          GO enrichmen and network analysisi to be performed.
# Input: Tibble with differentially expressed genes.
# Output: Edited file name.
extract_filename_go <- function(deg_file_tib){
  filename <- deg_file_tib$deg_file
  filename<-filename %>%
    str_remove('.tsv')
  filename<-sub(".*/", "", filename)
}



# Function name: post_process_GO_results
# Purpose: Assosiate file names with GO results.
# Input: GO results.
# Output: Named list.
post_process_GO_results <- function(GO_results){
  file_names <- GO_results[length(GO_results)] %>%
    unlist(use.names = FALSE)
  GO_results[length(GO_results)] <- NULL
  names(GO_results$result_GO_gene) <- file_names
  GO_results <- GO_results %>%
    flatten()
}



# Function name: DREM_main
# Purpose: Extract required data for executing DREM.
# Input: Pipeline input structure, wgcna_input.
# Output: None
```

```
DREM_main <- function(pipeline_input, wgcna_input, sig, exec){

  analysis_type <- pipeline_input %>%
    extract2('analysis_type')

  if(wgcna_input %>% str_detect('Sleuth')){
    map_dir <- pipeline_input %>%
      extract2('kallisto_output_dir') %>%
      paste0('Kallisto_quantifications/')

    dataset <- 'kallisto'
  }

  if(wgcna_input %>% str_detect('edgeR')){
    map_dir <- pipeline_input %>%
      extract2('star_genomeDir') %>%
      str_replace('genome_Dir', 'Feature_counts/')

    dataset <- 'Feature_counts'
  }

  # Set paths to DREM input data to be created.
  design_matrix <- pipeline_input %>%
    extract2('design_matrix')

  drem_time_series_input_path <- getwd() %>%
    paste0('/data/DREM/', analysis_type, '/')


  dir.create(drem_time_series_input_path, recursive = TRUE, showWarnings
= FALSE)

  drem_defaults_template <- pipeline_input %>%
    extract2('DREM') %>%
    paste0('/defaults.txt') %>%
    str_replace('//', '/')


  design_matrix <- design_matrix %>%
    read_csv(col_types = cols()) %>%
    dplyr::select(sample, condition, which_replicate)


  loaded_read_data <- load_read_data(map_dir, design_matrix, dataset)

  tmp <- rearrange_count_data(loaded_read_data,
pipeline_input$sample_covariates) %>%
    # reduce by sig hits
    mutate(
      reads = map(data, right_join, sig, by = 'target_id')
    )

  time_series_count_data <- tmp %>%
```

```
    write_DREM_time_series_data(., pipeline_input,
drem_time_series_input_path) %>%
    write_default_files(defaults_template = drem_defaults_template) %>%
    ungroup()


  DREM <- pipeline_input %>%
    extract2('DREM') %>%
    list.files(pattern = '*.jar', full.names = T)

  # Execute DREM script.
  DREM_command_line <- paste('java -mx1024M -jar', DREM, '-b')

  # Write batch DREM to script.
  DREM_execute <- time_series_count_data %>%
    dplyr::select(new_default_file_name) %>%
    mutate(
      command = map_chr(new_default_file_name,
                        ~paste(DREM_command_line, .x)
      ),
      file = str_replace(command, '^.* (.*)$', '\\1') %>%
        str_replace('.txt$', '_outfile.txt'),
      command_complete = paste(command, file)
    ) %>%
    dplyr::select(command_complete) %>%
    unlist(use.names = F)

  drem_script_location <- getwd() %>%
    paste0('/scripts/DREM_', pipeline_input %>%
extract2('analysis_type'), '.sh')

  drem_script <- drem_script_location %>%
    file()

  writeLines(DREM_execute, drem_script)

  close(drem_script)

  previous_dir <- getwd()
  setwd(pipeline_input$DREM)

  message('Currently running DREM. \n')

  for(i in seq_along(DREM_execute)){
    current <- DREM_execute[[i]] %>%
      str_extract('-b .*defaults.txt') %>%
      basename() %>%
      paste0('Generated defaults file: ', .)

    out <- DREM_execute[[i]] %>%
      stri_reverse() %>%
      gsub(pattern = ' .*', replacement = '') %>%
      stri_reverse() %>%
      basename()
```

```
    message(current)
    message('DREM configuration file: ', out)
    system(DREM_execute[[i]], show.output.on.console = T)
  }

  for(i in seq_along(DREM_execute)){
    current <- DREM_execute[[i]] %>%
      str_extract('-b .*defaults.txt') %>%
      basename()
    system(paste0('java -mx1024M -jar drem.jar'))

  }


  #if (exec){
  #  system(drem_script_location)
  #}
  setwd(previous_dir)
  return(tmp)

}




# Function name: load_read_data
# Purpose: Load count data.
# Input: Directory with reads, experimental design
#        matrix, and flag.
# Output: Loaded read data.
load_read_data <- function(map_dir, dm, dataset){
  if(dataset == 'Feature_counts'){
    SKIP <- 1
    TARGETS <- c(1, 2)
  } else{
    SKIP = 0
    TARGETS <- c('target_id', 'est_counts')
  }


  # manipulate read data to be input to DREM.
  reads <- list.files(map_dir, full.names = TRUE, recursive = T, pattern
= '.txt$') %>%
    str_replace(pattern = '//', '/') %>%
    tibble(file = .) %>%
    mutate(
      reads = map(file, ~read_tsv(.x, skip = SKIP, col_types = cols())
%>% dplyr::select(!!!TARGETS)
      )
    ) %>%
    bind_cols(dm)
}
```

```
clean_target_id <- function(data){
  data %>%
    dplyr::select(target_id = 1, counts = 2) %>%
    mutate(
      target_id = str_remove(target_id, '\\..*')
    )
}



# Function name: rearrange_count_data
# Purpose: Create data sets formatted for DREM.
# Input: Counts.
# Output: Time series count data.
rearrange_count_data <- function(reads, covars){

  reads2 <- reads %>%
    tidyr::extract(condition,
                   into = str_split(pattern = ', ', covars) %>% unlist(),
                   regex = '(.*) (.*) ([[:digit:]].*)') %>%
    mutate(
      reads = purrr::map(reads,
                         clean_target_id
      )
    )


  time_series_spread <- reads2 %T>%
    {
      # extract and sort numeric time series points.
      hour_vector <<- reads2 %>%
        use_series(hour) %>%
        unique() %>%
        str_remove(pattern = '[A-Za-z]') %>%
        as.numeric() %>%
        sort.int() %>%
        paste0('H', .)

    } %>%

    # Match hour vector element structures to the hour column in the
data.
    mutate(
      hour = hour %>%
        str_remove('[a-zA-Z]') %>%
        str_replace('^', 'H')
    ) %>%
    dplyr::select(-file, -sample) %>%

    # Associate each condition with a time series from the expression
data.
    unnest(reads) %>%
```

```
    group_by(genotype,
             condition,
             which_replicate,
             target_id) %>%
    spread(hour,
           counts) %>%
    ungroup() %>%
    dplyr::select(genotype,
           condition,
           which_replicate,
           target_id,
           hour_vector) %>%
    group_by(genotype,
             condition,
             which_replicate) %>%
    nest()

  return(time_series_spread)
}



# Function name: write_DREM_time_series_data
# Purpose: Write DREM-formatted data sets to files.
# Input: Reformatted counts, pipeline input structure,
#        path to each individual reformatted count data
#        set.
# Output: Reformatted counts.
write_DREM_time_series_data <- function(time_series_spread,
pipeline_input, drem_time_series_input_path){
  # create path to time series data.
  time_series_spread <- time_series_spread %>%
    mutate(
      file_name = paste(genotype, condition, which_replicate, sep = '_')
%>%
        paste0(drem_time_series_input_path, ., '.tsv')
    )
  # write time series counts to tsv files.
  time_series_spread %$%
    walk2(data,
          file_name,
          write_tsv)

  return(time_series_spread)
}





# Function name: write_default_files
# Purpose: Create DREM configuration file and write data.
# Input: Reformatted counts, and a configuration file template.
# Output: Reformatted counts nested on genotype.
```

```
write_default_files <- function(reformatted_counts, defaults_template){


  # load original defaults file from DREM2.
  defaults_file <- defaults_template %>%
    read.delim(row.names = NULL) %>%
    as_tibble() %>%
    mutate_if(is.factor, as.character)


  nest_on_genotype <- reformatted_counts %>%

    dplyr::select(genotype, condition, which_replicate, file_name) %>%
    group_by(genotype, condition) %>%
    nest() %>%
    dplyr::select(write_data = data) %>%
    ungroup() %>%

    mutate(
      new_default_file = map(write_data,
                             insert_DREM_input_to_defaults,
                             defaults_file
      ),
      new_default_file_name = pmap_chr(
        list(genotype, condition, write_data),
        write_new_default_file_name
      )
    )

  nest_on_genotype %$%
    walk2(new_default_file,
          new_default_file_name,
          write_tsv,
          col_names = FALSE)
  return(nest_on_genotype)

}





# Function name: insert_DREM_input_to_defaults
# Purpose: Create default files to execute DREM
#          for samples.
insert_DREM_input_to_defaults <- function(condition_data, defaults_file){

  # Max replicates.
  total_replicates <- condition_data %>%
    dplyr::select(which_replicate) %>%
    max()


  replicate_file <- condition_data %>%
```

```
    ungroup() %>%
    dplyr::select(file_name)

  defaults_file <- defaults_file %>%
    dplyr::select(a = 1, b = 2)

  defaults_file$b[3] <- replicate_file[1, 1] %>%
    pull()

  defaults_file$b[12] <- replicate_file[c(2:total_replicates), 1] %>%
    pull() %>%
    str_c(collapse = ',')

  return(defaults_file %>% unnest(b))
}



# Function name: write_new_default_file_name
# Purpose: Create a file name to write DREM defaults script.
# Input: Sample characteristics (genotype, condition).
# Output: Tibble for collecting DREM.
write_new_default_file_name <- function(genotype, condition, drem_data){

  file_name <- drem_data %>%
    ungroup() %>%
    dplyr::select(file_name) %>%
    first() %>%
    str_replace('.tsv$', 'defaults.txt') %>%
    first()

  return(file_name)
}




# Function name: mapping_network_analysis
# Purpose: Apply network analysis to each comparison dataset.
# Input: expr2.
# Output: Top genes or intersection with top GO results.
mapping_network_analysis <- function(expr2){
  expr2 %>%
    mutate(
      network_analsis = map2(clustering,
                             GO_results,
                             network_analysis)
    )
}
```

implement_network_analysis.R

```
# Function name: network_analysis
# Purpose: finds the network of differentialy expressed genes and collect
the hubbed genes of each comaprisions
# Input: adjacency matrix, expression data, Go results , Dynamic modules
# Output: network graph, most important genes
network_analysis <- function(expr2, GO_results){

  m <- expr2$adjacency
  source_node = c()
  target_node = c()
  correlation <- c()
  genes_names <- names(expr2$datExpr)
  i <- genes_names[1:dim(m)[1]]
  j <- i


  for(gene in i)
  {
    for(gen in j)
    {
      if(m[gene,gen] < 0.9999 & m[gene,gen] > 0.3){
        source_node <- c(source_node, gene)
        target_node <- c(target_node, gen)
        correlation <- c(correlation, m[gene,gen])
      }
    }
  }

  NetworkData <- data.frame(source_node, target_node, correlation)


  network_dataframe <- data.frame(source_node,target_node,correlation)


  # cluster membership info from WCGNA
  nodes <- as.data.frame(cbind(genes_names,  expr2$dynamic_Modules))
  rownames(nodes)<-NULL
  colnames(nodes) <- c("genes","cluster")

  # igraph_network dataset
  igraph_network_dataframe <- network_dataframe
  igraph_network_dataframe <- aggregate(igraph_network_dataframe[,3],
igraph_network_dataframe[,-3], sum)
  igraph_network_dataframe <-
igraph_network_dataframe[order(igraph_network_dataframe$source_node,
igraph_network_dataframe$target_node),]
  colnames(igraph_network_dataframe)[3] <- "weight"
  rownames(igraph_network_dataframe) <- NULL

  # igraph object:
  net <- graph.data.frame(igraph_network_dataframe, directed=F)


  # Generate colors base on clustering done by WGCNA:
```

```
  nodeIndex<-c()
  for(name in V(net)$name){
    nodeIndex<-c(nodeIndex,which(nodes$genes==name))
  }
  colors <- expr2$dynamic_Colors[nodeIndex]


  V(net)$color <- colors


  degree_nodes <- igraph::degree(net, mode ="total")
  V(net)$size <- degree_nodes


  #ching arrow width , label color, edge width etc.
  V(net)$label.color <- "black"
  E(net)$width <- E(net)$weight*2

  E(net)$arrow.size <- 10
  edge_width <-(E(net)$weight-mean(E(net)$weight))*5+1

  #ploting the graph

tkplot(net,edge.arrow.size=1,edge.curved=0,edge.width=edge_width,edge.col
or="gray80")

  #taking the cluster color
  cluster_colors<-unique(V(net)$color)

  #making an empty list of hubbed genes
  genes_hubbed<-list()

  #finding out the node centrality score of all the clusters and
therefore finding out the hubbed genes
  for(i in 1: length(unique(V(net)$color))){
    #finding nodes for the particlular cluster
    nodes_of_interest <- V(net)[which(V(net)$color == cluster_colors[i])]
    #finding the subgraph for the cluster nodes
    selgraph <- ego(net, order = 1, nodes =nodes_of_interest , mode =
"all",
                    mindist = 0)
    subgraph_cluster <- induced_subgraph(net,unlist(selgraph))

    #find the centrality score of each node in that cluster
    centarlity_score <- hub_score(subgraph_cluster , scale = TRUE,
weights = NULL,
                                  options = arpack_defaults)

    #find out the hubbed genes based on centrality score
    hubbed_genes <-
names(centarlity_score$vector[which(centarlity_score$vector >=
quantile(centarlity_score$vector,.95))])
    hubbed_genes<-as.data.frame(hubbed_genes)
    genes_hubbed[i]<-hubbed_genes
```

```
    rm(hubbed_genes)
    rm(nodes_of_interest)
    rm(selgraph)
    rm(selegoG)
    rm(centarlity_score)

  }

  hubbed_genes_in_total <- as.data.frame(levels(unlist(genes_hubbed)))
  colnames(hubbed_genes_in_total)<-"hubbed_genes"

  #selecting important genes on the basis of node degree
  top_node_dgree_genes<-
as.data.frame(names(V(net)[which(V(net)$size>=quantile(V(net)$size,0.95))
]))
  colnames(top_node_dgree_genes)<-"hubbed_genes"

  #merging the important genes from the node degree and hubbed genes and
finding top genes from Network Analysis
  top_genes<-merge(x = hubbed_genes_in_total, y = top_node_dgree_genes,
by = "hubbed_genes", all = TRUE) %>%
    unlist(use.names = FALSE)

  #finding the TOP hubbed genes frm the GO results and network anlysis
  hub_go_intersection <- intersect(GO_results, top_genes)

  if(length(hub_go_intersection) > 0){
    print('Hub genes intersected with top GO genes.')
    return(hub_go_intersection)
  } else{
    print('Hub genes do not intersect with top GO genes.')
    return(top_genes)
  }
}
```
